# Mathematical Characterization of Private and Public Immune Receptor Sequences

**DOI:** 10.1007/s11538-023-01190-z

**Published:** 2023-09-14

**Authors:** Lucas Böttcher, Sascha Wald, Tom Chou

**Affiliations:** 1 Department of Computational Science and Philosophy, Frankfurt School of Finance and Management, 60322 Frankfurt am Main, Germany; 2 Department of Computational Medicine, University of California, Los Angeles, 621 Charles E. Young Dr. S., Los Angeles, 90095-1766 CA USA; 3 Department of Medicine, University of Florida, Gainesville, 32610 FL USA; 4 Statistical Physics Group, Centre for Fluid and Complex Systems, Coventry University, Priory Street, Coventry, CV1 5FB UK; 5 Department of Mathematics, University of California, Los Angeles, 520 Portola Plaza, Los Angeles, 90095-1555 CA USA

**Keywords:** T cell repertoire, Diversity, Public/private clones, Overlap, Sampling

## Abstract

Diverse T and B cell repertoires play an important role in mounting effective immune responses against a wide range of pathogens and malignant cells. The number of unique T and B cell clones is characterized by T and B cell receptors (TCRs and BCRs), respectively. Although receptor sequences are generated probabilistically by recombination processes, clinical studies found a high degree of sharing of TCRs and BCRs among different individuals. In this work, we use a general probabilistic model for T/B cell receptor clone abundances to define “publicness” or “privateness” and information-theoretic measures for comparing the frequency of sampled sequences observed across different individuals. We derive mathematical formulae to quantify the mean *and the variances* of clone richness and overlap. Our results can be used to evaluate the effect of different sampling protocols on abundances of clones within an individual as well as the commonality of clones across individuals. Using synthetic and empirical TCR amino acid sequence data, we perform simulations to study expected clonal commonalities across multiple individuals. Based on our formulae, we compare these simulated results with the analytically predicted mean and variances of the repertoire overlap. Complementing the results on simulated repertoires, we derive explicit expressions for the richness and its uncertainty for specific, single-parameter truncated power-law probability distributions. Finally, the information loss associated with grouping together certain receptor sequences, as is done in spectratyping, is also evaluated. Our approach can be, in principle, applied under more general and mechanistically realistic clone generation models.

## Introduction

A major component of the adaptive immune system in most jawed vertebrates is the repertoire of B and T lymphocytes. A diverse immune repertoire allows the adaptive immune system to recognize a wide range of pathogens (Xu et al. 2020). B and T cells develop from common lymphoid progenitors (CLPs) that originate from hematopoietic stem cells (HSCs) in the bone marrow. B cells mature in the bone marrow and spleen while developing T cells migrate to the thymus where they undergo their maturation process. After encountering an antigen, naive B cells may get activated and differentiate into antibody-producing plasma cells, which are essential for humoral (or antibody-mediated) immunity. In recognizing and eliminating infected and malignant cells, T cells contribute to cell-mediated immunity of adaptive immune response.

T-cell receptors bind to antigenic peptides (or epitopes) that are presented by major histocompatibility complex (MHC) molecules on the surface of antigen-presenting cells (APCs). T cells that each carry a type of TCR mature in the thymus and undergo V(D)J recombination, where variable (V), diversity (D), and joining (J) gene segments are randomly recombined (Alt et al. 1992; Travers et al. 1997). The receptors are heterodimeric molecules and mainly consist of an $\alpha$ and a $\beta$ chain while only a minority, about 1–10% (Girardi 2006), of TCRs consists of a $\delta$ and a $\gamma$ chain. The TCR $\alpha$ and $\gamma$ chains are made up of VJ and constant (C) regions. Additional D regions are present in $\beta$ and $\gamma$ chains. During the recombination process, V(D)J segments of each chain are randomly recombined with additional insertions and deletions. After recombination, only about 5% or even less (Yates 2014) of all generated TCR sequences are selected based on their ability to bind to certain MHC molecules (“positive selection”) and to not trigger autoimmune responses (“negative selection”). These naive T cells are then exported from the thymus into peripheral tissue where they may interact with foreign peptides that are presented by APCs. The selection process as well as subsequent interactions are specific to an individual.

The most variable parts of TCR sequences are the complementary determining regions (CDRs) 1, 2, and 3, located within the V region, among which the CDR3$\beta$ is the most diverse (Abbas et al. 2021). Therefore, the number of distinct receptor sequences, the richness *R*, of TCR repertoires is typically characterized in terms of the richness of CDR3$\beta$ sequences. Only about 1% of T cells express two different TCR$\beta$ chains (Davodeau et al. 1995; Padovan et al. 1995; Schuldt and Binstadt 2019), whereas the proportion of T cells that express two different TCR$\alpha$ chains may be as high as 30% (Rybakin et al. 2014; Schuldt and Binstadt 2019).

B cells can also respond to different antigens via different B cell receptors (BCRs) that are comprised of heavy and light chains. As with TCRs, the mechanism underlying the generation of a diverse pool of BCRs is VDJ recombination in heavy chains and VJ recombination in light chains. Positive and negative selection processes sort out about 90% of all BCRs that react too weakly or strongly with certain molecules (Tussiwand et al. 2009). As a result of the various recombination and joining processes and gene insertions and deletions, the practical theoretical maximum repertoire size $\Omega _{0}$ of the variable region of BCR and TCR receptors can be $\sim 10^{14}{-}10^{20}$ (Davis and Bjorkman 1988; Venturi et al. 2008; Zarnitsyna et al. 2013; Lythe et al. 2016). This value is comparable to the possible number of amino acid sequences of typical length $\sim 11{-}12$. However, many of these sequences are not viable, are removed through thymic selection, or are have such low probability occuring that they are never expected to be produced in an organism’s lifetime. Thus, the effective number of TCR variable regions that are produced and that can contribute to the organism’s repertoire size, $\Omega$, should be much less than $\Omega _{0}$. Estimating the true size of BCR and TCR repertoires realized in an organism is challenging since the majority of such analyses are based on small blood samples, leading to problems similar to the “unseen species” problem in ecology (Laydon et al. 2015). Nonetheless, the number of unique TCRs realized in organisms has been estimated to be about $10^6$ for mice (Casrouge et al. 2000) and about $10^8$ for humans (Soto et al. 2020). B-cell repertoire size for humans is estimated to be $10^8{-}10^9$ (DeWitt et al. 2016). These values are significantly smaller than $\Omega _{0}$ and might be used as an effective $\Omega$.

Each pool of BCR and TCR sequences realized in one organism *i* can be seen as a subset ${\mathcal {U}}_i$ of the set of all possible species-specific sequences ${\mathcal {S}}$. Sequences that occur in at least two different organisms *i* and *j* (i.e., sequences that are elements of ${\mathcal {U}}_i\cap {\mathcal {U}}_j$) are commonly referred to as “public” sequences (Laydon et al. 2015) while “private” sequences occur only in one of the individuals tested. The existence of public TCR$\beta$ sequences has been established in several previous works (Putintseva et al. 2013; Robins et al. 2010; Shugay et al. 2013; Soto et al. 2020). More recently, a high degree of shared sequences has been also observed in human BCR repertoires (Briney et al. 2019; Soto et al. 2019).

The notions of public and private clonotypes have been loosely defined. Some references use the term “public sequence” to refer to those sequences that “are *often* shared between individuals” (Shugay et al. 2013) or “shared across individuals” (Greiff et al. 2017). Recently, Elhanati et al. (2018) and Ruiz Ortega et al. (2023) have formulated a mathematical and statistical framework to quantify “publicness” and “privateness.” Building on these works, we derive a set of measures that enable us to quantify immune repertoire properties, including the expected total richness, the expected numbers of public and private clones, *and their variances* (confidence levels), all expressed in terms of the general set of clone generation probabilities or clone populations. One of our metrics is the expected “*M*-overlap” or “*M*-publicness,” defined as the expected number of clones that appear in samples drawn from *M* separate individuals. This quantity is a clinically interpretable limit of the expected “sharing number” defined in Elhanati et al. (2018). Similarly, we define *M*-private clones as clones that are not shared by all *M* individuals, i.e., occurring in at most $M-1$ individuals.

In the next section, we first give an overview of the mathematical concepts that are relevant to characterize TCR and BCR distributions. We then formulate a statistical model of receptor distributions in Sect. 3. In Sects. 3.1 and 3.2, we derive quantities associated with receptor distributions in single organisms and across individuals, respectively. We will primarily focus on the overlap of repertoires across individuals and on the corresponding confidence intervals that can be used to characterize “public” and “private” sequences of immune repertoires. Formulae we derived are listed in Table 1. In Sect. 4, we use synthetic and empirical TCR amino acid sequence data and perform simulations to compare theoretical predictions of repertoire overlaps between different individuals with corresponding observations. Finally, when analyzing empirical sequence data, one may use continuous approximations (Elhanati et al. 2018; Ruiz Ortega et al. 2023) and averaging (i.e., coarse-graining) methods that change the information content in the underlying dataset. Coarse-graining of TCR and BCR data may also be a result of the employed sequencing techniques (Gorski et al. 1994; Fozza et al. 2017). In Sect. 6, we therefore briefly discuss the information loss associated with analyzing processed cell data. We discuss our results and conclude our paper in Sect. 7. Our source codes are publicly available at GitLab (2022).

**Table 1:** Table of mathematical results

Measure	Description	$\textbf{n}$-representation	$\textbf{p}$-representation
${\mathbb {E}}[R]$	Individual richness	$\sum _{k=1}^{N}\sum _{i=1}^{\Omega }\mathbbm {1}(n_{i},k)$	$\sum _{i=1}^{\Omega }\rho _{i}$
${\mathbb {E}}[R^{2}]$	$2^{\textrm{nd}}$ moment of richness	$\left[ \sum _{k=1}^{N}\sum _{i=1}^{\Omega }\mathbbm {1}(n_{i},k)\right] ^{2}$	$\sum _{i=1}^{\Omega }\rho _{i}+\sum _{j\ne i}^{\Omega }\rho _{ij}$
${\mathbb {E}}[R_{\textrm{s}}]$	Mean sampled richness	$\sum _{i=1}^{\Omega }\sigma _{i} = \Omega - {1\over {N \atopwithdelims ()S}}\sum _{i=1}^{\Omega } {N-n_{i}\atopwithdelims ()S}$	$\sum _{i=1}^{\Omega } \rho _{i}(S)$
${\mathbb {E}}[R_{\textrm{s}}^{2}]$	$2^{\textrm{nd}}$ moment, sampled richness	$\sum _{i=1}^{\Omega }\sigma _{i}+\sum _{j\ne i}^{\Omega }\sigma _{ij}$	$\sum _{i=1}^{\Omega }\rho _{i}(S)+\sum _{j\ne i}^{\Omega }\rho _{ij}(S)$
${\mathbb {E}}[R^{(M)}]$	Mean group richness	$\sum _{k\ge 1}\sum _{i=1}^{\Omega }\mathbbm {1}\left( \textstyle {\sum _{m=1}^{M}}n_{i}^{(m)}, k\right)$	$\sum _{i=1}^{\Omega }{\tilde{\rho }}_{i}$
${\mathbb {E}}\left[ (R^{(M)})^{2}\right]$	$2^{\textrm{nd}}$ mom., grp richness	$\left[ \sum _{k\ge 1}\sum _{i=1}^{\Omega } \mathbbm {1}\left( \textstyle {\sum _{m=1}^{M}}n_{i}^{(m)}, k\right) \right] ^{2}$	$\sum _{i}^{\Omega }{\tilde{\rho }}_{i} + \sum _{i\ne j}^{\Omega }{\tilde{\rho }}_{ij}$
$\textrm{var}[R^{(M)}]$	Variance of grp richness	0	$\sum \limits _{i\ne j}^{\Omega }{\tilde{\rho }}_{ij} + (1-\sum \limits _{i=1}^{\Omega } {\tilde{\rho }}_{i})\sum \limits _{i=1}^{\Omega }{\tilde{\rho }}_{i}$
${\mathbb {E}}{[}R_{\textrm{s}}^{(M)}]$	Mean sampled grp richness	$\sum _{i=1}^{\Omega }{\tilde{\sigma }}_{i}$	$\sum _{i=1}^{\Omega }{\tilde{\rho }}_{i}(S)$
${\mathbb {E}}\left[ (R_{\textrm{s}}^{(M)})^{2}\right]$	$2^{\textrm{nd}}$ mom., sampled grp richness	$\sum _{i=1}^{\Omega }{\tilde{\sigma }}_{i} + \sum _{i\ne j}^{\Omega }{\tilde{\sigma }}_{ij}$	$\sum _{i=1}^{\Omega }{\tilde{\rho }}_{i}(S)+ \sum _{i\ne j}^{\Omega }{\tilde{\rho }}_{ij}(S)$
${\mathbb {E}}[K^{(M)}]$	Expected *M*-overlap	$\sum _{i=1}^{\Omega }\prod _{m=1}^{M}\sum _{k^{(m)}\ge 1}\!\mathbbm {1}(n_{i}^{(m)}\!,k^{(m)})$	$\sum _{i=1}^{\Omega }\prod _{m=1}^{M}\rho _{i}^{(m)}$
${\mathbb {E}}\left[ (K^{(M)})^{2}\right]$	$2^{\textrm{nd}}$ moment, *M*-overlap	$\left[ \sum \limits _{i=1}^{\Omega }\prod \limits _{m=1}^{M}\sum \limits _{k^{(m)}\ge 1}\! \mathbbm {1}(n_{i}^{(m)}\!,k^{(m)})\right] ^{2}$	$\sum \limits _{i=1}^{\Omega }\prod \limits _{m=1}^{M}\rho _{i}^{(m)} +\sum \limits _{i\ne j}^{\Omega }\prod \limits _{m=1}^{M}\rho _{ij}^{(m)}$
${\mathbb {E}}[K_{\textrm{s}}^{(M)}]$	Sampled *M*-overlap	$\sum _{i=1}^{\Omega }\prod _{m=1}^{M}\sigma _{i}^{(m)}$	$\sum _{i=1}^{\Omega }\prod _{m=1}^{M}\rho _{i}^{(m)}(S)$
${\mathbb {E}}[(K_{\textrm{s}}^{(M)})^{2}]$	$2^{\textrm{nd}}$ mom., sampled *M*-overlap	$\sum \limits _{i=1}^{\Omega }\prod \limits _{m=1}^{M}\sigma _{i}^{(m)}(S) +\sum \limits _{i\ne j}^{\Omega }\prod \limits _{m=1}^{M}\sigma _{ij}^{(m)}(S)$	$\sum \limits _{i=1}^{\Omega }\prod \limits _{m=1}^{M}\rho _{i}^{(m)}(S) +\!\sum \limits _{i\ne j}^{\Omega }\prod \limits _{m=1}^{M}\!\rho _{ij}^{(m)}(S)$

We list our main mathematical derivations and expressions for richness and overlap, unsampled and sampled, in both the $\textbf{n}$-representation and the $\textbf{p}$-representation. The component probabilities $\rho _{i}^{(m)}$, $\rho _{ij}^{(m)}$, ${\tilde{\rho }}_{i}$, ${\tilde{\rho }}_{ij}$, $\sigma _{i}^{(m)}$, $\sigma _{ij}^{(m)}$, ${\tilde{\sigma }}_{i}$, and ${\tilde{\sigma }}_{ij}$ are given in Eqs. (6), (8), (13), (16), (24), (25), (26), and (27), respectively. The component probabilities $\rho _{i}^{(m)}(S)$, $\rho _{ij}^{(m)}(S)$, ${\tilde{\rho }}_{i}(S)$, ${\tilde{\rho }}_{ij}(S)$ associated with sampled quantities in the $\textbf{p}$-representation are given by Eqs. (37), (38), (39), and (40), respectively

## Mathematical Concepts

**Fig. 1:**
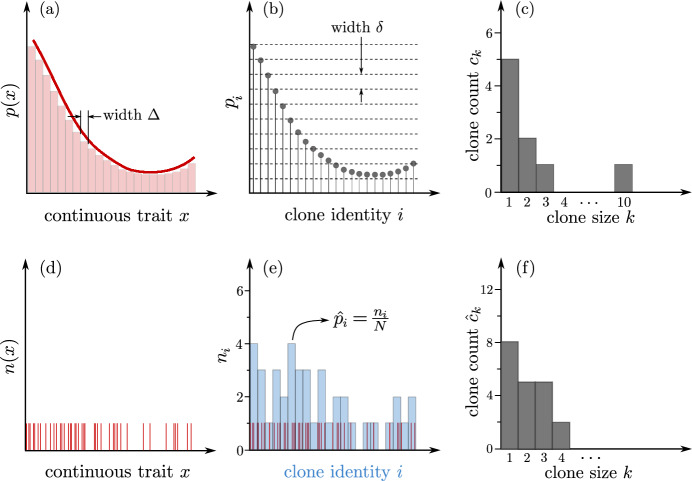
Sampling from a continuous distribution, described in terms of an underlying probability density *p*(*x*) and number density *n*(*x*). The probability density *p*(*x*) (solid red line) and the Riemann sum approximation to the probability (red bars of width $\Delta$) are shown in panel (**a**). The probability that a trait in the interval $[x,x+\textrm{d}x)$ arises is $p(x)\textrm{d}x$. As shown in (**b**), this distribution can be discretized directly by the intervals $[i\Delta ,(i+1)\Delta )$ (red bars) defining discrete traits and their associated probabilities $p_i$ (see Eq. (1)). The probabilities $p_i$ can be transformed into clone counts $c_k$ (the number of identities *i* that are represented by *k* individuals) using Eq. (2), which are shown in (**c**). A finite sample of a population described by *p*(*x*) yields the binary outcome shown in (**d**). In this example, the total number of samples is $N=41$ and since the trait space *x* is continuous, the probability that the exact same trait arises in more than one sample is almost surely zero. Light blue bars in panel (**e**) represent number counts $n_i$ binned according to $\Delta$. The probabilities ${\hat{p}}_i=n_i/N$ provide an approximation of $p_i$. Clone counts for the empirical ${\hat{p}}_i$ are calculated according to Eq. (3) and shown in (**f**) (Color figure online)

Although receptor sequences and cell counts are discrete quantities, using continuous functions to describe their distribution may facilitate the mathematical analysis of the quantities that we derive in the subsequent sections. For example, a continuous approximation (i.e., a “density-of-states approximation”) has been used to characterize the number of T cell receptor sequences possible within a continuous range of generation probabilities (Murugan et al. 2012; Elhanati et al. 2018; Ruiz Ortega et al. 2023). Another instance of a continuous cell statistics approximation involves employing power laws to describe the rank-abundance curves associated with immune repertoires (see, e.g., Gaimann et al. 2020). We therefore briefly review some elementary concepts associated with continuous distributions and their discretization.

Let *p*(*x*) be the *probability density* associated with the distribution of traits, as depicted in Fig. 1a. The probability that a certain trait occurs in $[x,x+\textrm{d}x)$ is $p(x)\,\textrm{d}x$. The corresponding discretized distribution elements are

1 $$\begin{aligned} p_i{:}{=}\int \nolimits _{i \Delta }^{(i+1)\Delta }\!\!\! p(x)\,\textrm{d}x, \end{aligned}$$

where $\Delta$ is the discretization step size of the support of *p*(*x*). If we discretize the values of probabilities, the number of clones with a certain relative frequency $p_i$ is given by the *clone count*

2 $$\begin{aligned} c_k{:}{=}\sum _i \mathbbm {1}\left( k\delta \le p_{i}<(k+1)\delta \right) , \end{aligned}$$

where the indicator function $\mathbbm {1}=1$ if its argument is satisfied and 0 otherwise. As shown in Fig. 1b, the parameter $\delta$ defines an interval of frequency values and modulates the clone-count binning. Figures 1b, c show how $p_i$ and $c_k$ are constructed from a continuous distribution *p*(*x*). If *p*(*x*) is not available from data or a model, an alternative representative starts with the number density *n*(*x*), which can be estimated by sampling a process which follows *p*(*x*). The probability that a continuous trait *x* is drawn twice from a continuous distribution *p*(*x*) is almost surely zero. Hence, the corresponding number counts *n*(*x*) are either 1 if $X\in [x,x+\textrm{d}x)$ (i.e., if trait *X* is sampled) or 0 otherwise, as shown in Fig. 1d, e. We say that *X* is of *clonotype* *i* if $X\in [i \Delta ,(i+1)\Delta )$ ($1\le i \le \Omega$) and we use $n_i$ to denote the number of cells of clonotype *i*. Then, if $\Omega$ denotes the effective number of different clonotypes, the total T-cell (or B-cell) population is $N\equiv \sum _{i=1}^\Omega n_i$. The relative empirical abundance of clonotype *i* is thus ${\hat{p}}_i=n_i/N$ (see Fig. 1e), satisfying the normalization condition $\sum _i {\hat{p}}_i=1$. Besides the simple discrete estimate ${\hat{p}}_i=n_i/N$, one can also reconstruct *p*(*x*) from ${\textbf {n}}=\{n_{i}\}$ using methods such as kernel density estimation. The corresponding empirical clone count derived from the number representation $n_{i}$ is defined as

3 $$\begin{aligned} {\hat{c}}_{k} {:}{=}\sum _{i=1}^{\Omega }\mathbbm {1}(n_{i},k) \end{aligned}$$

and shown in Fig. 1f. The indicator function $\mathbbm {1}(a,b)$ with arguments $a,b\in \mathbb {Z}_{\ge 0}$ is equal to 1 if $a=b$ and 0 otherwise. Clone counts can be used to describe T cell repertoires, especially if clone identities are not important. Simple birth-death-immigration models can also be cast in terms of, e.g., expected clone counts ${\mathbb {E}}[{\hat{c}}_{k}(t)]$ (Goyal et al. 2015; Lewkiewicz et al. 2019).

## Whole Organism Statistical Model

Using the mathematical quantities defined above, we develop a simple statistical model for BCR and TCR sequences distributed among individuals. Although our model is applicable to both BCR and TCR sequences, we will primarily focus on the characterization of TCRs for simplicity. B cells undergo an additional process of somatic hypermutation and class switching leading to a more dynamic evolution of the more diverse B cell repertoire (Elhanati et al. 2015). By focusing on naive T cells, we can assume their populations are generated by the thymus via a single, simple effective process.

**Fig. 2:**
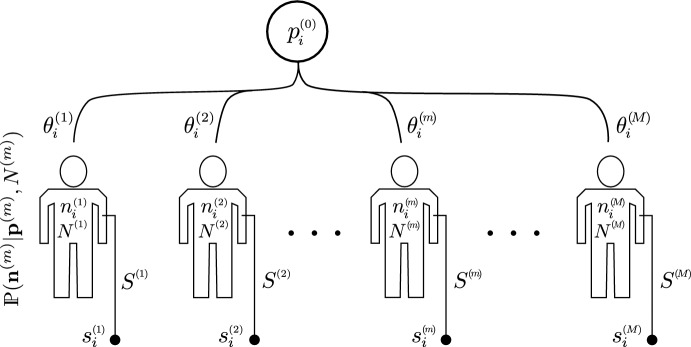
Schematic of sampling of multiple species from multiple individuals. A central process produces (through V(D)J recombination) TCRs. Individuals select for certain TCRs resulting in population $n_{i}^{(m)}$ of T cells with receptor *i* in individual *m*, for a total T-cell count $N^{(m)} = \sum _{i}^{\Omega }n_{i}^{(m)}$. The selection of TCR *i* by individual *m* (in their individual thymuses) is defined by the parameter $\theta _i^{(m)}$ which gives an effective probability $p_{i}^{(m)}\equiv \theta _{i}^{(m)} p_{i}^{(0)}$. A sample with cell numbers $S^{(m)} \ll N^{(m)}$ is drawn from individual *m* and sequenced to determine $s_{i}^{(m)}$, the number of cells of type *i* in the subsample drawn from individual *m*

Assume a common universal recombination process (see Fig. 2) in T-cell development that generates a cell carrying TCR of type $1\le i\le \Omega _{0}$ with probability $p_{i}^{(0)}$. Here, $\Omega _{0}\gg \Omega$ is the theoretical number of ways the full TCR sequence can be constructed which is itself much larger than the effective number $\Omega$ that appears in an individual after thymic selection. Although each new T cell produced carries TCR *i* with probability $p_{i}^{(0)}$, many sequences *i* are not realized given thymic selection (that eliminates $\sim 98\%$ of them), the finite number of T cells produced over a lifetime (Travers et al. 1997; Yates 2014; Lythe et al. 2016), or the extremely low generation probability of some clones. These effects are invoked to truncate $\Omega _{0}$ to $\Omega \ll \Omega _{0}$. However, we will see in Sect. 5, explicit scaling relastionships for the limit $\Omega \gg 1$ can be found for general power-law ordered probabilities $p_{i}$.

Besides VDJ recombination (Slabodkin et al. 2021), thymic selection and subsequent death, activation, and proliferation occur differently across individuals $1\le m \le M$ and may be described by model parameters $\theta _{i}^{(m)}$. Such a model translates the fundamental underlying recombination process into a population of $n_{i}^{(m)}$ T cells with TCR *i* and total population $N^{(m)}= \sum _{i=1}^{\Omega }n_{i}^{(m)}$ in individual *m*. The connection between $p_{i}^{(0)}, \theta _{i}^{(m)}$ and $n_{i}^{(m)}, N^{(m)}$ might be described by dynamical models, deterministic or stochastic, such as those presented in Dessalles et al. (2022).

At any specific time, individual *m* will have a cell population configuration $\textbf{n}^{(m)} \equiv (n_{1}^{(m)}, n_{2}^{(m)}, \ldots , n_{\Omega }^{(m)})$ with probability $\mathbb {P}(\textbf{n}^{(m)}\vert \varvec{\theta }^{(m)},N^{(m)})$. Each individual can be thought of as a biased sample from all cells produced via the universal probabilities $p_{i}^{(0)}$. We can approximate individual probabilities $p_{i}^{(m)} \equiv \theta _{i}^{(m)}p_{i}^{(0)},\, 1\le i \le \Omega$, where the number of effective TCRs $\Omega$ for individual *m* might have as upper bound $\Omega \sim 10^{14}$, if, for example, we are considering just the CDR3 region of the $\beta$ chain. Assuming a time-independent model (*e.g*, a model in steady-state), we can describe the probability of a T-cell population $\textbf{n}^{(m)}$ in individual *m* by a multinomial distribution over individual probabilities $\textbf{p}^{(m)} \equiv \{p_{i}^{(m)}\}$:

4 $$\begin{aligned} \mathbb {P}(\{\textbf{n}^{(m)}\}\vert \{\textbf{p}^{(m)},N^{(m)}\}) = N^{(m)}! \prod _{i=1}^{\Omega }{[p_{i}^{(m)}]^{n_{i}^{(m)}}\over n_{i}^{(m)}!}, \end{aligned}$$

where $\sum _{i=1}^{\Omega }n_{i}^{(m)} \equiv N^{(m)}$ and $\sum _{i=1}^{\Omega }p_{i}^{(m)} \equiv 1$. Thus, each individual can be thought of as a “sample” of the “universal” thymus. Neglecting genetic relationships amongst individuals, we can assume them to be independent with individual probabilities $p_{i}^{(m)}$. These are the probabilities that a randomly drawn cell from individual *m* is a cell of clone *i*. Repeated draws (with replacement) would provide the samples for the estimator ${\hat{p}}_{i}^{(m)}= n_{i}^{(m)}/N^{(m)}$, assuming $n_{i}^{(m)}$ are counted and $N^{(m)}$ is also known or estimated. This representation allows us to easily express the probabilities of any configuration $\textbf{n}^{(m)}$ analytically. A dynamical model for $n_{i}^{(m)}$ cannot be directly described by our simple probabilities $p_{i}^{(m)}$. A mechanistically more direct model could incorporate the production rate of clone *i* T cells from the thymus, the proliferation and apoptosis rates of clone *i* cells, and interactions manifested as, e.g., carrying capacity as model parameters. Probability distributions for $\textbf{n}^{(m)}$, as a function of birth, death, and immigration rates, have been found in Dessalles et al. (2018) and can also be used, instead of Eq. (4), to construct probabilities.

### Single Individual Quantities

First, we focus on quantities intrinsic to a single individual organism; thus, we can suppress the “$m=1$” label. Within an individual, we can use clone counts to define measures such as the richness

5 $$\begin{aligned} R(\textbf{n}) {:}{=}\sum _{i=1}^\Omega \mathbbm {1}(n_{i}\ge 1) =\sum _{k \ge 1} \sum _{i=1}^{\Omega } \mathbbm {1}(n_{i}, k) \equiv \sum _{k\ge 1}{\hat{c}}_{k}, \end{aligned}$$

where ${\hat{c}}_{k}\equiv \sum _{i=1}^{\Omega } \mathbbm {1}(n_{i}, k)$ is defined in Eq. (3) (the number of clones that are of size *k*). Other diversity/entropy measures such as Simpson’s indices, Gini indices, etc. Rempala and Seweryn (2013) and Xu et al. (2020) can also be straightforwardly defined. Given the clone populations $\textbf{n}$, the individual richness can be found by direct enumeration of Eq. (5).

We can also express the richness in terms of the underlying probabilities $\textbf{p}$ associated with the individual by first finding the probability $\rho _{i}$ that a type-*i* cell appears at all among the *N* cells within said individual. This probability is

6 $$\begin{aligned} \rho _{i} \equiv \mathbb {P}(n_{i}\ge 1\vert \textbf{p}, N) = 1-(1-p_{i})^{N} \end{aligned}$$

and corresponds to that of a binary outcome, either appearing or not appearing. Higher order probabilities like $\rho _{ij}$ (both *i*- and *j*-type cells appearing in a specific individual) can be computed using the marginalized probability

7 $$\begin{aligned} \mathbb {P}(n_{i}, n_{j} \vert \textbf{p}, N) = {N!\, p_{i}^{n_{i}}p_{j}^{n_{j}} (1-p_{i}-p_{j})^{N-n_{i}-n_{j}} \over n_{i}!\, n_{j}!\, (N-n_{i}-n_{j})!} \end{aligned}$$

to construct

8 $$\begin{aligned} \begin{aligned} \rho _{ij}&\equiv \mathbb {P}(n_{i}, n_{j} \ge 1\vert \textbf{p},N) \\&= 1+(1-p_i-p_j)^{N} -(1-p_{i})^{N}-(1-p_{j})^{N}. \end{aligned} \end{aligned}$$

Higher moments can be straightforwardly computed using quantities such as

9 $$\begin{aligned} \begin{aligned} \rho _{ijk}&\equiv \mathbb {P}(n_{i}, n_{j}, n_{k} \ge 1 \vert \textbf{p}, N) \\ \,&= 1 -(1-p_{i}-p_{j}-p_{k})^{N}\!-\!\!\sum _{\ell =i,j,k}\!\!(1-p_{\ell })^{N} +\!\!\!\!\sum _{q\ne \ell =i,j,k}\!\!\!(1-p_{q}-p_{\ell })^{N}. \qquad \end{aligned} \end{aligned}$$

These expressions arise when we compute the moments of *R* [defined by Eq. (5)] in terms of the probabilities $\textbf{p}$. Using the single-individual multinomial probability $\mathbb {P}(\textbf{n}\vert \textbf{p},N)$ (Eq. (4)) allows us to express moments of the richness in a single individual in terms of the underlying system probabilities $\textbf{p}$. The first two are given by

10 $$\begin{aligned} \begin{aligned} \mathbb {E}[R(\textbf{p})]&= \sum _{\textbf{n}}\sum _{i=1}^{\Omega } \mathbbm {1}(n_{i}\ge 1) \mathbb {P}(\textbf{n}\vert \textbf{p}, N) = \sum _{i=1}^{\Omega }\mathbb {P}(n_{i}\ge 1\vert \textbf{p}, N)=\sum _{i=1}^{\Omega }\rho _{i}, \\ \mathbb {E}[R^{2}(\textbf{p})]&= \sum _{\textbf{n}} \Big [\sum _{i=1}^{\Omega }\mathbbm {1}(n_i\ge 1)\Big ]^{2} \mathbb {P}(\textbf{n}\vert \textbf{p}, N)\\ \,&= \sum _{i,j=1}^{\Omega }\mathbb {P}(n_{i},n_{j} \ge 1\vert \textbf{p}, N) \equiv \mathbb {E}[R]+ \sum _{j\ne i}^{\Omega }\rho _{ij}. \end{aligned} \end{aligned}$$

### Multi-individual Quantities

Here, we consider the distribution $\textbf{n}^{(m)}$ across different individuals and construct quantities describing group properties. For example, the combined richness of all TCR clones of *M* individuals is defined as

11 $$\begin{aligned} R^{(M)}(\{\textbf{n}^{(m)}\}) {:}{=}\sum _{k\ge 1}\sum _{i=1}^{\Omega }\mathbbm {1}\Big (\textstyle {\sum \limits _{m=1}^{M}}n_{i}^{(m)}\!, k\Big ). \end{aligned}$$

To express the expected multi-individual richness in terms of the underlying individual systems probabilities $\textbf{p}^{(m)}$, we weight Eq. (11) over the *M*-individual probability

12 $$\begin{aligned} \mathbb {P}_{M}(\{\textbf{n}^{(m)}\}\vert \{\textbf{p}^{(m)}, N^{(m)}\}) \equiv \prod _{m=1}^{M}\mathbb {P}(\{\textbf{n}^{(m)}\}\vert \{\textbf{p}^{(m)}, N^{(m)}\}), \end{aligned}$$

and sum over all allowable $\textbf{n}^{(m)}$. For computing the first two moments of the total-population richness in terms of $\textbf{p}^{(m)}$, we will make use of the marginalized probability ${\tilde{\rho }}_{i}$ that clone *i* appears in at least one of the *M* individuals

13 $$\begin{aligned} \begin{aligned} {\tilde{\rho }}_{i}&\equiv \mathbb {P}\Big (\textstyle {\sum \limits _{m=1}^{M}}n_{i}^{(m)} \ge 1 \left| \{ \textbf{p}^{(m)}, N^{(m)} \}\Big )\right. = 1-\mathbb {P}\left( n_{i}^{(m)}=0\,\, \forall \, m\right) \\&= 1-\prod _{m=1}^{M}\left( 1-p_{i}^{(m)}\right) ^{N^{(m)}}. \end{aligned} \end{aligned}$$

Note that ${\tilde{\rho }}_{i} >\prod _{m=1}^{M}\rho _{i}^{(m)}$ describes the probability that a type *i* cell occurs at all in the total population, while $\prod _{m=1}^{M}\rho _{i}^{(m)}$ describes the probability that a type *i* cell appears in each of the *M* individuals.

We will also need the joint probability that clones *i* and *j* both appear in at least one of the *M* individuals $\mathbb {P}\big (\sum _{m=1}^{M} n_{i}^{(m)} \ge 1, \sum _{\ell =1}^{M}n_{j}^{(\ell )} \ge 1\vert \{\textbf{p}^{(m)}, N^{(m)}\}\big )$, which we can decompose as

14 $$\begin{aligned} \begin{aligned}&\mathbb {P}\big (\textstyle {\sum \limits _{m=1}^{M}}n_{i}^{(m)}\ge 1, \textstyle {\sum \limits _{\ell =1}^{M}}n_{j}^{(\ell )} \ge 1\left| \{\textbf{p}^{(m)},N^{(m)}\}\big )\right. \\&\hspace{2.4cm} = 1-\mathbb {P}\big (\textstyle {\sum \limits _{m=1}^{M}}n_{i}^{(m)} \ge 1, \textstyle {\sum \limits _{\ell =1}^{M}}n_{j}^{(\ell )} =0 \left| \{\textbf{p}^{(m)}, N^{(m)}\}\big )\right. \\&\hspace{3cm} - \mathbb {P}\big (\textstyle {\sum \limits _{m=1}^{M}}n_{i}^{(m)} =0, \textstyle {\sum \limits _{\ell =1}^{M}}n_{j}^{(\ell )} \ge 1 \left| \{\textbf{p}^{(m)}, N^{(m)}\}\big )\right. \\&\hspace{3cm}- \mathbb {P}\big (\textstyle {\sum \limits _{m=1}^{M}}n_{i}^{(m)} = \textstyle {\sum \limits _{\ell =1}^{M}}n_{j}^{(\ell )}=0 \left| \{\textbf{p}^{(m)}, N^{(m)}\}\big )\right. . \end{aligned} \end{aligned}$$

Upon using Eqs. (4) and (7), we find

15 $$\begin{aligned} \begin{aligned}&\mathbb {P}\big (\textstyle {\sum \limits _{m=1}^{M}}n_{i}^{(m)} \ge 1, \textstyle {\sum \limits _{\ell =1}^{M}}n_{j}^{(\ell )} =0 \left| \{ \textbf{p}^{(m)}, N^{(m)}\}\big )\right. \\&\quad = \prod _{m=1}^{M}\!\big (1-p_{i}^{(m)}\big )^{N^{(m)}}\!\!\!\! - \prod _{m=1}^{M}\!\big (1-p_{i}^{(m)}\!\!\!-p_{j}^{(m)}\big )^{N^{(m)}}, \end{aligned} \end{aligned}$$

allowing us to rewrite Eq. (14) as

16 $$\begin{aligned} \begin{aligned} {\tilde{\rho }}_{ij}&\equiv \mathbb {P}\big (\textstyle {\sum \limits _{m=1}^{M}}n_{i}^{(m)}\ge 1, \textstyle {\sum \limits _{\ell =1}^{M}}n_{j}^{(\ell )}\!\ge 1\left| \{\textbf{p}^{(m)}, N^{(m)}\}\big )\right. \\&= 1 -\!\prod _{m=1}^{M}\!\big (1-p_{i}^{(m)}\big )^{N^{(m)}}\!\!\!\!- \prod _{m=1}^{M}\!\big (1-p_{j}^{(m)}\big )^{N^{(m)}} \\ \,&\quad + \prod _{m=1}^{M}\!\big (1-p_{i}^{(m)}\!\!\!-p_{j}^{(m)}\big )^{N^{(m)}}\!\!\!\!\!. \end{aligned} \end{aligned}$$

Note also that ${\tilde{\rho }}_{ij} > \prod _{m=1}^{M}\rho _{ij}^{(m)}$.

Using the above definitions, we can express the mean total-population richness as

17 $$\begin{aligned} \begin{aligned} \mathbb {E}[R^{(M)}(\{\textbf{p}^{(m)}\})]&= \sum _{\textbf{n}^{(m)}}\sum _{i=1}^{\Omega } \sum _{k\ge 1}\mathbbm {1}\Big (\textstyle {\sum \limits _{\ell =1}^{M}}n_{i}^{(\ell )}\!,k\Big ) \prod _{m=1}^{M}\mathbb {P}(\{\textbf{n}^{(m)}\}\vert \{\textbf{p}^{(m)}, N^{(m)}\}) \\ \,&= \sum _{i=1}^{\Omega } \mathbb {P}\Big (\textstyle {\sum \limits _{m=1}^{M}}n_{i}^{(m)} \ge 1\left| \{\textbf{p}^{(m)}, N^{(m)}\}\Big )\right. \\ \,&= \sum _{i=1}^{\Omega }\left[ 1-\prod _{m=1}^{M}(1-p_{i}^{(m)})^{N^{(m)}}\right] \equiv \sum _{i=1}^{\Omega } {\tilde{\rho }}_{i} \\ \,&= \Omega - \sum _{i=1}^{\Omega }\prod _{m=1}^{M}(1-p_{i}^{(m)})^{N^{(m)}} \\ \,&\approx \Omega - \sum _{i=1}^{\Omega } e^{-\sum _{m=1}^{M}p_{i}^{(m)}N^{(m)}}\!\!\!, \end{aligned} \end{aligned}$$

where the last approximation holds for $p_{i}^{(m)}\ll 1, N^{(m)}\gg 1$. The second moment of the total *M*-population richness can also be found in terms of $\mathbb {E}[R^{(M)}]$ and Eq. (16),

18 $$\begin{aligned} \begin{aligned} \mathbb {E}\Big [\left( R^{(M)}(\{\textbf{p}^{(m)}\})\right) ^{2}\Big ]&= \sum _{\{\textbf{n}^{(M)}\}}\left[ \sum _{i=1}^{\Omega }\mathbbm {1}\Big (\textstyle {\sum \limits _{m=1}^{M}}n_{i}^{(m)}\ge 1\Big )\right] ^{2} \prod _{m=1}^{M}\mathbb {P}\left( \{\textbf{n}^{(m)} \}\vert \{\textbf{p}^{(m)}, N^{(m)}\}\right) \\&= \sum _{i,j=1}^{\Omega }\mathbb {P}\Big (\textstyle {\sum \limits _{m=1}^{M}}n_{i}^{(m)}\ge 1, \textstyle {\sum \limits _{\ell =1}^{M}}n_{j}^{(\ell )}\ge 1 \left| \{\textbf{p}^{(m)}, N^{(m)}\}\Big )\right. \\&= \sum _{i=1}^{\Omega }\mathbb {P}\Big (\textstyle {\sum \limits _{m=1}^{M}}n_{i}^{(m)}\ge 1 \left| \{\textbf{p}^{(m)}, N^{(m)}\}\Big )\right. \\&\quad + \sum _{i\ne j}^{\Omega } \mathbb {P}\Big (\textstyle {\sum \limits _{m=1}^{M}}n_{i}^{(m)}\ge 1, \textstyle {\sum \limits _{\ell =1}^{M}}n_{j}^{(\ell )}\ge 1 \left| \{\textbf{p}^{(m)}, N^{(m)}\}\Big )\right. \\&= \, \mathbb {E}[R^{(M)}(\{\textbf{p}^{(m)}\})] + \sum _{i\ne j}^{\Omega }{\tilde{\rho }}_{ij}. \end{aligned} \end{aligned}$$

Given $\textbf{n}^{(m)}$ of all individuals, we can also easily define the number of distinct TCR clones that appear in all of *M* randomly selected individuals, the “*M*-overlap” or “*M*-publicness”

19 $$\begin{aligned} K^{(M)}(\{\textbf{n}^{(m)}\}){:}{=}\sum _{i=1}^{\Omega }\prod _{m=1}^{M}\sum _{k^{(m)}\ge 1} \!\mathbbm {1}(n_{i}^{(m)}\!,k^{(m)}). \end{aligned}$$

**Fig. 3:**
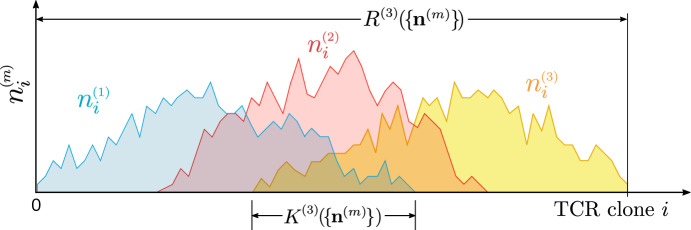
Three individuals with overlapping cell number distributions $n_{i}^{(m)}, m=1,2,3$. The richness $R^{(3)}$ is the total number of distinct TCRs found across all individuals, and is defined in Eq. (11). The overlap $K^{(3)}$ is the number of TCR clones found in all three individuals, as defined in Eq. (19). For visual simplicity, the set of clones *i* present in each individual are drawn to be contiguous. When considering subsampling of cells from each individual, $K^{(M)}$ will be reduced since some cell types *i* will be lost. The corresponding values, $s_{i}^{(m)}$, $K_{\textrm{s}}^{(M)}$, and $R_{\textrm{s}}^{(M)}$ can be constructed from Eqs. (23) and (24) reflecting the losses from subsampling

Figure 3 provides a simple example of three individuals each with a contiguous distribution of cell numbers $n_{i}^{(m)}$ that overlap.

As with Eqs. (5) and (10), we can express the overlap in terms of the underlying individual probabilities $\textbf{p}^{(m)}$ by weighting Eq. (19) by the *M*-population probability $\prod _{m=1}^{M} \mathbb {P}(\textbf{n}^{(m)}\vert \textbf{p}^{(m)},N^{(m)})$ (see Eq. (4)) to find

20 $$\begin{aligned}{} & {} \begin{aligned} \mathbb {E}[K^{(M)}(\{\textbf{p}^{(m)}\})]&= \sum _{\{\textbf{n}^{(m)}\}}\left[ \sum _{i=1}^{\Omega }\prod _{m=1}^{M} \mathbbm {1}(n_{i}^{(m)}\ge 1)\right] \mathbb {P}_{M}(\{\textbf{n}^{(m)}\}\vert \{\textbf{p}^{(m)}, N^{(m)}\})\\&= \sum _{i=1}^{\Omega }\prod _{m=1}^{M}\mathbb {P}(n_{i}^{(m)}\ge 1\vert \{\textbf{p}^{(m)}, N^{(m)}\})\\&= \sum _{i=1}^{\Omega }\prod _{m=1}^{M}\left[ 1-\left( 1-p_{i}^{(m)}\right) ^{N^{(m)}}\right] \equiv \sum _{i=1}^{\Omega }\prod _{m=1}^{M}\rho _{i}^{(m)} \end{aligned} \end{aligned}$$

21 $$\begin{aligned}{} & {} \begin{aligned} \,&\mathbb {E}\Big [\left( K^{(M)}(\{\textbf{p}^{(m)}\})\right) ^{2}\Big ]\\ \,&\hspace{7mm} = \sum _{\{\textbf{n}^{(m)}\}} \left[ \sum _{i=1}^{\Omega }\prod _{m=1}^{M} \mathbbm {1}(n_{i}^{(m)}\ge 1)\right] ^{2} \mathbb {P}_{M}(\{\textbf{n}^{(m)}\}\vert \{\textbf{p}^{(m)}, N^{(m)}\}) \\ \,&\hspace{7mm} = \sum _{i,j=1}^{\Omega }\prod _{m=1}^{M} \mathbb {P}(n_{i}^{(m)},n_{j}^{(m)}\ge 1\vert \{\textbf{p}^{(m)}, N^{(m)}\})\\ \,&\hspace{7mm} = \sum _{i=1}^{\Omega }\prod _{m=1}^{M} \left[ 1-(1-p_{i}^{(m)})^{N^{(m)}}\right] \\ \,&\hspace{11mm}+\sum _{j\ne i}^{\Omega }\prod _{m=1}^{M}\! \left[ 1+(1-p_{i}^{(m)}\!\!-p_{j}^{(m)})^{N^{(m)}}-(1-p_{i}^{(m)})^{N^{(m)}}\!\!\!\!-(1-p_{j}^{(m)})^{N^{(m)}}\right] \\ \,&\hspace{7mm} \equiv \mathbb {E}[K^{(M)}(\{\textbf{p}^{(m)}\})]+\sum _{j\ne i}^{\Omega }\prod _{m=1}^{M}\rho _{ij}^{(m)}. \end{aligned} \end{aligned}$$

The expected number of clones shared among all *M* individuals, $\mathbb {E}[K^{(M)}]$, provides a natural measure of *M*-overlap. Clearly, $\mathbb {E}[K^{(M)}]<\mathbb {E}[K^{(M')}]$ if $M>M'$. As with *M*-publicness, we can identify the expected *M*-privateness as $\Omega -\mathbb {E}\left[ K^{(M)}\right]$, the expected number of clones that are not shared by all *M* individuals, i.e., that occur in at most $M-1$ individuals. This “privateness” is related to a multi-distribution generalization of the “dissimilarity probability” of samples from two different discrete distributions (Hampton and Lladser 2012). Variations in *M*-overlap associated with a certain cell-type distribution are captured by the variance $\textrm{var}\left[ K^{(M)}\right] =\mathbb {E}\left[ (K^{(M)})^{2}\right] -\mathbb {E}\left[ K^{(M)}\right] ^2$. If the total number of sequences $\Omega$ is very large, parallelization techniques (see Sect. 4) should be employed to evaluate the term $\sum _{j\ne i}^{\Omega }\prod _{m=1}^{M}\rho _{ij}^{(m)}$ in $\mathbb {E}\left[ (K^{(M)})^{2}\right]$.

A more specific definition of overlap or privateness may be that a clone must appear in at least some specified fraction of *M* tested individuals. To find the probability that $M_{i} \le M$ individuals share at least one cell of a single type *i*, we use the Poisson binomial distribution describing independent Bernoulli trials on individuals with different success probabilities $\rho _{i}^{(m)} \equiv \rho (n_{i}^{(m)} \ge 1)$:

22 $$\begin{aligned} \mathbb {P}(M_{i}\vert \{p_{i}^{(m)}\}) = \sum _{A\in F_{M_{i}}}\prod _{m \in A}\rho _{i}^{(m)} \prod _{\ell \in A^\textrm{c}}(1-\rho _{i}^{(\ell )}), \end{aligned}$$

where $F_{M_{i}}$ is the set of all subsets of $M_{i}$ integers that can be selected from the set $(1,2,3,\ldots ,M)$ and $A^{\textrm{c}}$ is the complement of *A*. Equation (22) gives a probabilistic measure of the prevalence of TCR *i* across *M* individuals. For example, one can use it to define a mean frequency $\mathbb {E}[M_{i}]/M$. One can evaluate Eq. (22) recursively or using Fourier transforms, particularly for $M < 20$ (Chen and Liu 1997; Hong 2013).

### Subsampling

The results above are described in terms of the entire cell populations $\textbf{n}^{(m)}$ or their intrinsic generation probabilities $\textbf{p}^{(m)}$. In practice, one cannot measure $n_{i}^{(m)}$ or even $N^{(m)}$ in any individual *m*. Rather, we can only sample a much smaller number of cells $S^{(m)} \ll N^{(m)}$ from individual *m*, as shown in Fig. 2. Within this subsample from individual *m*, we can count the number $s_{i}^{(m)}$ of type-*i* cells. Since only subsamples are available, we wish to define quantities such as probability of occurrence, richness, and overlap in terms of the cell counts $\textbf{s}^{(m)} \equiv \{s_{i}^{(m)}\}$ in the sample extracted from an individual. Quantities such as *sampled* richness and overlap can be defined in the same way except with $\textbf{s}^{(m)}$ as the underlying population configuration. To start, first assume that the cell count $\textbf{n}$ in a specific individual is given. If that individual has *N* cells of which *S* are sampled, the probability of observing the population $\textbf{s}= \{s_{1}, s_{2}, \ldots , s_{\Omega }\}$ in the sample is given by (assuming all cells are uniformly distributed and randomly subsampled at once, without replacement) (Chao and Lin 2012)

23 $$\begin{aligned} \begin{aligned} \mathbb {P}(\textbf{s}\vert \textbf{n}, S, N)&= {1\over {N \atopwithdelims ()S}} \prod _{i=1}^{\Omega }{n_{i} \atopwithdelims ()s_{i}},\quad \sum _{i=1}^{\Omega }s_{i} = S. \end{aligned} \end{aligned}$$

The probability that cell type *j* appears in the sample from an individual with population $\textbf{n}$ can be found by marginalizing over all $s_{j\ne i}$, giving

24 $$\begin{aligned} \begin{aligned} \sigma _{i} \equiv \mathbb {P}(s_{i}\ge 1 \vert \textbf{n}, S, N)&\displaystyle = 1-{{N-n_{i}\atopwithdelims ()S}\over {N\atopwithdelims ()S}}. \end{aligned} \end{aligned}$$

This result can be generalized to more than one TCR clone present. For example, the probability that both clones *i* and *j* are found in a sample is

25 $$\begin{aligned} \begin{aligned} \sigma _{ij}\equiv \mathbb {P}(s_{i}, s_{j} \ge 1 \vert \textbf{n}, S, N)&= \displaystyle 1+{{N-n_{i}-n_{j}\atopwithdelims ()S}\over {N\atopwithdelims ()S}}- {{N-n_{i}\atopwithdelims ()S}\over {N\atopwithdelims ()S}}-{{N-n_{j}\atopwithdelims ()S}\over {N\atopwithdelims ()S}}. \end{aligned} \end{aligned}$$

Using Eq. (23) as the probability distribution, we can also find the probability that clone *i* appears in any of the *M* $S^{(m)}$-sized samples

26 $$\begin{aligned} \begin{aligned} {\tilde{\sigma }}_{i} \equiv \mathbb {P}\Big (\textstyle {\sum \limits _{m=1}^{M}}s_{i}^{(m)}\ge 1 \left| \{\textbf{n}^{(m)}, S^{(m)}, N^{(m)}\}\Big )\right.&\displaystyle = 1-\prod _{m=1}^{M}{{N^{(m)}-n_{i}^{(m)}\atopwithdelims ()S^{(m)}}\over {N^{(m)}\atopwithdelims ()S^{(m)}}}, \end{aligned} \end{aligned}$$

and the joint probabilities that clones *i* and *j* appear in any sample

27 $$\begin{aligned} \begin{aligned} {\tilde{\sigma }}_{ij}&\equiv \mathbb {P}\Big (\textstyle {\sum \limits _{m=1}^{M}}s_{i}^{(m)}\ge 1, \textstyle {\sum \limits _{\ell =1}^{M}}s_{j}^{(\ell )} \ge 1 \left| \{\textbf{n}^{(m)}, S^{(m)}, N^{(m)}\}\Big )\right. \\&= \displaystyle 1+\prod _{m=1}^{M}\!{{N^{(m)}-n_{i}^{(m)}-n_{j}^{(m)}\atopwithdelims ()S^{(m)}}\over {N^{(m)}\atopwithdelims ()S^{(m)}}}- \prod _{m=1}^{M}\!{{N^{(m)}-n_{i}^{(m)}\atopwithdelims ()S^{(m)}}\over {N^{(m)}\atopwithdelims ()S^{(m)}}} -\prod _{m=1}^{M}\!{{N^{(m)}-n_{j}^{(m)}\atopwithdelims ()S^{(m)}} \over {N^{(m)}\atopwithdelims ()S^{(m)}}}. \end{aligned} \end{aligned}$$

Quantities such as richness and publicness *measured within samples* from the group can be analogously defined in terms of clonal populations $\textbf{s}^{(m)}$:

28 $$\begin{aligned}{} & {} R_{\textrm{s}}(\textbf{s}){:}{=}\sum _{k\ge 1}\sum _{i=1}^{\Omega } \mathbbm {1}(s_{i}, k), \end{aligned}$$

29 $$\begin{aligned}{} & {} R_{\textrm{s}}^{(M)}(\{\textbf{s}^{(m)}\}) {:}{=}\sum _{k\ge 1}\sum _{i=1}^{\Omega }\mathbbm {1}\big (\textstyle {\sum \limits _{m=1}^{M}}s_{i}^{(m)}, k\big ), \end{aligned}$$

and

30 $$\begin{aligned} K_{\textrm{s}}^{(M)}(\{\textbf{s}^{(m)}\}){:}{=}\sum _{i=1}^{\Omega }\prod _{m=1}^{M} \sum _{k^{(m)}\ge 1} \mathbbm {1}(s_{i}^{(m)},k^{(m)}). \end{aligned}$$

For a given $\textbf{n}^{(m)}$, these quantities can be first averaged over the sampling distribution Eq. (23) to express them in terms of $\textbf{n}^{(m)}$ and to explicitly reveal the effects of random sampling. The first two moments of $R_{\textrm{s}}$, $R^{(M)}_{\textrm{s}}$, and $K_{\textrm{s}}^{(M)}$ expressed in terms of $\textbf{n}^{(m)}$ can be easily found by weighting Eqs. (28),  (29), and (30) by $\mathbb {P}(\textbf{s}\vert \textbf{n}, S, N)$ and $P^{(M)} = \prod _{m=1}^{M}\mathbb {P}(\textbf{s}^{(m)} \vert \textbf{n}^{(m)}, S^{(m)}, N^{(m)})$:

31 $$\begin{aligned}{} & {} \begin{aligned} \mathbb {E}[R_{\textrm{s}}(\textbf{n})]&= \,\Omega - {1\over {N \atopwithdelims ()S}}\sum _{i=1}^{\Omega } {N-n_{i}\atopwithdelims ()S}\equiv \sum _{i=1}^{\Omega } \sigma _{i},\\ \mathbb {E}\big [\big (R_{\textrm{s}}(\textbf{n})\big )^{2}\big ]&= \, \mathbb {E}[R_{\textrm{s}}(\textbf{n})]+\sum _{i\ne j}^{\Omega }\sigma _{ij} \end{aligned} \end{aligned}$$

32 $$\begin{aligned}{} & {} \begin{aligned} \,&\mathbb {E}\big [R_{\textrm{s}}^{(M)}(\{\textbf{n}^{(m)}\})\big ]\\ \,&\hspace{6mm} =\sum _{\{\textbf{s}^{(m)}\}}\sum _{i=1}^{\Omega } \mathbbm {1}\Big (\textstyle {\sum \limits _{m=1}^M} s_{i}^{(m)}\ge 1\Big ) \prod _{m=1}^{M}\mathbb {P}(\{\textbf{s}^{(m)}\} \vert \{\textbf{n}^{(m)}\!, S^{(m)}\!, N^{(m)}\})\\ \,&\hspace{6mm} = \sum _{i=1}^{\Omega }\mathbb {P}\Big (\textstyle {\sum \limits _{m=1}^M} s_{i}^{(m)}\ge 1 \left| \{\textbf{n}^{(m)}\!, S^{(m)}\!, N^{(m)}\}\Big )\right. \\ \,&\hspace{6mm} = \Omega -\sum _{i=1}^\Omega \prod _{m=1}^M {{N-n_{i}^{(m)}\atopwithdelims ()S}\over {N\atopwithdelims ()S}} \equiv \sum _{i=1}^\Omega {\tilde{\sigma }}_{i}, \\ \,&\mathbb {E}\Big [\big (R_{\textrm{s}}^{(M)}(\{\textbf{n}^{(m)}\})\big )^{2}\Big ]\\ \,&\hspace{8mm} =\sum _{\{\textbf{n}^{(m)}\}}\left[ \sum _{i=1}^{\Omega } \mathbbm {1}\Big (\textstyle {\sum \limits _{m=1}^M} s_{i}^{(m)}\ge 1\Big )\right] ^{2} \prod _{m=1}^{M}\mathbb {P}(\{\textbf{s}^{(m)}\} \vert \{ \textbf{n}^{(m)}, S^{(m)}, N^{(m)}\}) \\ \,&\hspace{8mm}= \sum _{i,j=1}^{\Omega }\prod _{m=1}^{M}\mathbb {P} \Big (\textstyle {\sum \limits _{m=1}^M} s_{i}^{(m)}, \textstyle {\sum \limits _{\ell =1}^M} s_{j}^{(m)}\ge 1 \left| \{\textbf{n}^{(m)}, S^{(m)}, N^{(m)}\}\Big )\right. \\ \,&\hspace{8mm} = \mathbb {E}\big [R_\textrm{s}^{(M)}(\{\textbf{n}^{(m)}\})\big ] + \!\sum _{i\ne j=1}^\Omega \!{\tilde{\sigma }}_{ij}, \end{aligned} \end{aligned}$$

33 $$\begin{aligned}{} & {} \begin{aligned} \hspace{3mm} \mathbb {E}\big [K_{\textrm{s}}^{(M)}(\{\textbf{n}^{(m)}\})\big ]&\, \\ \,&\hspace{-20mm} =\! \sum _{\{\textbf{s}^{(m)}\}} \sum _{i=1}^{\Omega }\prod _{m=1}^{M}\!\mathbbm {1}(s_{i}^{(m)}\ge 1) \mathbb {P}(\textbf{s}^{(m)} \vert \{\textbf{n}^{(m)}\!, S^{(m)}\!, N^{(m)}\})\\ \,&\hspace{-20mm} = \sum _{i=1}^{\Omega }\prod _{m=1}^{M} \!\mathbb {P}(s_{i}^{(m)}\ge 1 \vert \{\textbf{n}^{(m)}\!, S^{(m)}\!, N^{(m)}\})\\ \,&\hspace{-20mm} = \sum _{i=1}^{\Omega } \prod _{m=1}^{M}\!\left[ 1-\frac{{N^{(m)}-n_{i}^{(m)}\atopwithdelims ()S^{(m)}}}{{N^{(m)}\atopwithdelims ()S^{(m)}}}\right] \equiv \sum _{i=1}^{\Omega } \prod _{m=1}^{M}\!\sigma _{i}^{(m)},\\ \mathbb {E}\Big [\big (K_{\textrm{s}}^{(M)}(\{\textbf{n}^{(m)}\})\big )^{2}\Big ]&\, \\ \,&\hspace{-20mm} = \sum _{\{\textbf{s}^{(m)}\}}\left[ \sum _{i=1}^{\Omega } \prod _{m=1}^{M} \mathbbm {1}(s_{i}^{(m)}\ge 1)\right] ^{2}\prod _{m=1}^{M} \mathbb {P}(\{\textbf{s}^{(m)}\} \vert \{\textbf{n}^{(m)}\!\!, S^{(m)}\!\!, N^{(m)}\}) \\ \,&\hspace{-20mm} = \sum _{i,j=1}^{\Omega }\prod _{m=1}^{M} \mathbb {P}(s_{i}^{(m)},s_{j}^{(m)}\ge 1 \vert \{\textbf{n}^{(m)}\!\!, S^{(m)}\!\!, N^{(m)}\}) \\ \,&\hspace{-20mm} \equiv \mathbb {E}\big [K_\textrm{s}^{(M)}(\{\textbf{n}^{(m)}\})\big ] +\sum _{i\ne j}^{\Omega } \prod _{m=1}^{M}\sigma _{ij}^{(m)}. \end{aligned} \end{aligned}$$

All of the above quantities can also be expressed in terms of the underlying probabilities $\textbf{p}^{(m)}$ rather than the population configurations $\textbf{n}^{(m)}$. To do so, we can further weight Eqs. (31), (32), and (33) over the probability Eq. (4) to render these quantities in terms of the underlying probabilities $\textbf{p}^{(m)}$. However, we can also first convolve Eq. (23) with the multinomial distribution in Eq. (4) (suppressing the individual index *m*)

34 $$\begin{aligned} \begin{aligned} \mathbb {P}(\textbf{s}\vert \textbf{p}, S, N)&= \sum _{\textbf{n}} \mathbb {P}(\textbf{s}\vert \textbf{n}, S, N) \mathbb {P}(\textbf{n}\vert \textbf{p}, N), \end{aligned} \end{aligned}$$

along with the implicit constraints $\sum _{i=1}^{\Omega }n_{i} \equiv N$ and $\sum _{i=1}^{\Omega }s_{i} = S$ to find

35 $$\begin{aligned} \begin{aligned} \mathbb {P}(\textbf{s}\vert \textbf{p}, S)&= S! \prod _{i=1}^{\Omega } {p_{i}^{s_{i}}\over s_{i}!},\quad \sum _{i=1}^{\Omega }s_{i} = S, \end{aligned} \end{aligned}$$

which is a multinomial distribution identical in form to $\mathbb {P}(\textbf{n}\vert \textbf{p}, N)$ in Eq. (4), except with $\textbf{n}$ replaced by $\textbf{s}$ and *N* replaced by *S*. Uniform random sampling from a multinomial results in another multinomial. Thus, if we use the full multi-individual probability

36 $$\begin{aligned} \mathbb {P}_{M}(\{\textbf{s}^{(m)}\} \vert \{\textbf{p}^{(m)}\!,S^{(m)}\}) \equiv \prod _{m=1}^{M}\mathbb {P}(\{\textbf{s}^{(m)}\}\vert \{\textbf{p}^{(m)}\!, S^{(m)}\}) \end{aligned}$$

to compute moments of the sampled richness and publicness, they take on the same forms as the expressions associated with the whole-organism quantities. For example, in the $\textbf{p}$ representation, the probability that clone *i* appears in the sample from individual *m* is

37 $$\begin{aligned} \rho _{i}^{(m)}(S)\equiv \mathbb {P}(s_{i}^{(m)}\ge 1 \vert \{\textbf{p}^{(m)}\!, S^{(m)}\}) = 1-(1-p_{i}^{(m)})^{S^{(m)}}\!\!, \end{aligned}$$

in analogy with Eq. (6), while the two-clone joint probability in the sampled from individual *m* becomes

38 $$\begin{aligned} \begin{aligned} \rho _{ij}^{(m)}(S)&\equiv \mathbb {P}(s^{(m)}_{i}, s^{(m)}_{j} \ge 1\vert \{\textbf{p}^{(m)}\!,S^{(m)}\})\\&= 1+(1-p^{(m)}_{i}\!\!-p_{j}^{(m)})^{S^{(m)}}\!\!\! -(1-p_{i}^{(m)})^{S^{(m)}}\!\!\!-(1-p_{j}^{(m)})^{S^{(m)}}, \end{aligned} \end{aligned}$$

in analogy with Eq. (8). Similarly, for the overlap quantities, in analogy with Eqs. (13) and (16), we have

39 $$\begin{aligned}{} & {} \begin{aligned} {\tilde{\rho }}_{i}(S)&\equiv \mathbb {P}\Big (\textstyle {\sum \limits _{m=1}^{M}}s_{i}^{(m)} \ge 1 \left| \{ \textbf{p}^{(m)}, S^{(m)} \}\Big )\right. = 1-\mathbb {P}\left( s_{i}^{(m)}=0\,\, \forall \, m\right) \\&= 1-\prod _{m=1}^{M}\left( 1-p_{i}^{(m)}\right) ^{S^{(m)}}. \end{aligned} \end{aligned}$$

40 $$\begin{aligned}{} & {} \begin{aligned} {\tilde{\rho }}_{ij}(S)&\equiv \mathbb {P}\Big (\textstyle {\sum \limits _{m=1}^{M}}s_{i}^{(m)}\ge 1, \textstyle {\sum \limits _{\ell =1}^{M}}s_{j}^{(\ell )} \ge 1\left| \{\textbf{p}^{(m)}, S^{(m)}\}\Big ) \right. \\&= 1-\!\!\prod _{m=1}^{M}\!\left( 1-p_{i}^{(m)}\right) ^{S^{(m)}}\!\!\!\!-\!\prod _{m=1}^{M}\! \left( 1-p_{j}^{(m)}\right) ^{S^{(m)}}\\&\quad \ + \prod _{m=1}^{M}\!\left( 1-p_{i}^{(m)}\!\!\!-p_{j}^{(m)}\right) ^{S^{(m)}}\!\!. \end{aligned} \end{aligned}$$

The expressions for the sampled moments $\mathbb {E}[R_{\textrm{s}}(\textbf{p})]$, $\mathbb {E}[R_{\textrm{s}}^{2}(\textbf{p})]$, $\mathbb {E}\big [R_\textrm{s}^{(M)}(\{\textbf{p}^{(m)}\})\big ]$, $\mathbb {E}\big [\big (R_\textrm{s}^{(M)}(\{\textbf{p}^{(m)}\})\big )^{2}\big ]$, $\mathbb {E}[K_\textrm{s}^{(M)}(\{\textbf{p}^{(m)}\})]$, and $\mathbb {E}\big [\big (K_\textrm{s}^{(M)}(\{\textbf{p}^{(m)}\})\big )^{2}\big ]$ follow the same form as their unsampled counterparts given in Eqs. (10), (17), (18), (20), and (21), except with $\rho _{i}^{(m)}$, $\rho _{ij}^{(m)}$, ${\tilde{\rho }}_{i}$, and ${\tilde{\rho }}_{ij}$ replaced by their $\rho _{i}^{(m)}(S)$, $\rho _{ij}^{(m)}(S)$, ${\tilde{\rho }}_{i}(S)$, and ${\tilde{\rho }}_{ij}(S)$ counterparts. This simplifying property arises because of the conjugate nature of the multinomial distributions (4), (35), and (34).

In addition to simple expressions for the moments of $K_\textrm{s}^{(M)}$, we can also find expressions for the probability distribution over the values of $K_{\textrm{s}}^{(M)}$. In terms of $\textbf{n}^{(m)}$, since the probability that $s_{i}^{(m)}\ge 1$ in the samples from all $1\le m \le M$ individuals is $\sigma _{i} \equiv \prod _{m=1}^{M} \mathbb {P}(s_{i}^{(m)}\ge 1\vert \{\textbf{n}^{(m)}, S^{(m)}, N^{(m)}\}) = \prod _{m=1}^{M}\sigma _{i}^{(m)}$, the probability that exactly *k* clones are shared by all *M* samples is

41 $$\begin{aligned} \mathbb {P}(K^{(M)}_{\textrm{s}} = k \vert \{\sigma _{i}^{(m)}\}) = \sum _{A\in F_{k}}\prod _{i \in A}\Big (\prod _{m=1}^{M}\sigma _{i}^{(m)}\Big ) \prod _{j\in A^\textrm{c}}\Big [1-\prod _{m=1}^{M}\sigma _{j}^{(m)}\Big ], \end{aligned}$$

where $F_{k}$ is the set of all subsets of *k* integers that can be selected from the set $\{1,2,3,\ldots , K^{(M)}\}$ and $A^{\textrm{c}}$ is the complement of *A*. Equation (41) is the Poisson binomial distribution, but this time the underlying success probabilities $\prod _{m=1}^{M}\sigma _{i}^{(m)}$ across all *M* individuals vary with TCR clone identity *i*.

Finally, inference of individual measures from subsamples can be formulated. One can use the sampling likelihood function $\mathbb {P}(\textbf{s}\vert \textbf{n}, S, N)$, Bayes’ rule, and the multinomial (conjugate) prior $\mathbb {P}(\textbf{n}\vert \textbf{p}, N)$ to construct the posterior probability of $\textbf{n}$ *given* a sampled configuration $\textbf{s}$:

42 $$\begin{aligned} \begin{aligned} \mathbb {P}(\textbf{n}\vert \textbf{s}, S, N, \textbf{p})&= \frac{\mathbb {P}(\textbf{s}\vert \textbf{n}, S, N) \mathbb {P}(\textbf{n}\vert \textbf{p}, N)}{\sum _{\textbf{n}} \mathbb {P}(\textbf{s}\vert \textbf{n}, S, N) \mathbb {P}(\textbf{n}\vert \textbf{p}, N)}. \end{aligned} \end{aligned}$$

The normalization in Eq. (42) has already been found in Eqs. (34) and (35). Thus, we find the posterior

43 $$\begin{aligned} \mathbb {P}(\textbf{n}\vert \textbf{s}, S, N, \textbf{p}) = (N-S)! \prod _{i=1}^{\Omega }\frac{p_{i}^{n_{i}-s_{i}}}{(n_{i}-s_{i})!},\quad \sum _{i=1}^{\Omega }(n_{i}-s_{i}) = N-S \end{aligned}$$

in terms of the hyperparameters $\textbf{p}$. Using this posterior, we can calculate the expectation of the whole organism richness $R=\sum _{k\ge 1}\sum _{i=1}^{\Omega } \mathbbm {1}(n_{i}, k)$,

44 $$\begin{aligned} \mathbb {E}[R(\textbf{s}, \textbf{p})] = \Omega - \sum _{j\vert s_{j}=0}\left( 1-p_{j}\right) ^{N-S}, \end{aligned}$$

which depends on the sampled configuration only through the sample-absent clones *j*. Bayesian methods for estimating overlap between two populations from samples have also been recently explored (Larremore 2019).

**Fig. 4:**
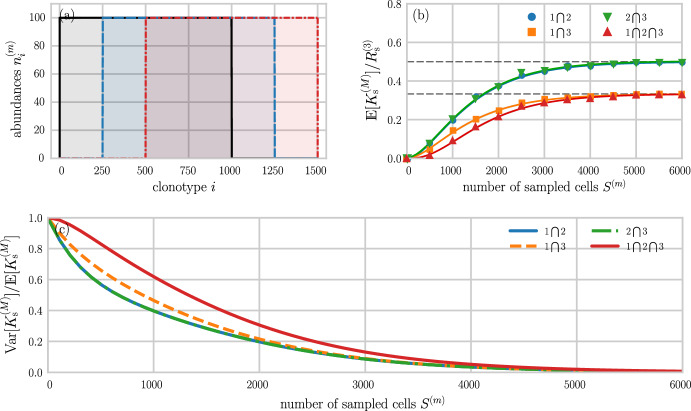
Sampling from shifted uniform distributions. **a** Synthetic TCR or BCR distributions of $M=3$ individuals. The distributions in individuals 1, 2, and 3 are indicated by solid black, dashed blue, and dash-dotted red lines, respectively. Each individual has $10^{5}$ cells uniformly distributed across 1000 clones (100 cells per clone). The sampled group richness $R_\textrm{s}^{(3)}$ is 1500. **b** Samples of size $S^{(m)}$ have been generated to compute the relative overlaps between individuals 1 and 2 (blue disks), 2 and 3 (green inverted triangles), 1 and 3 (orange squares), and 1–3 (red triangles). The solid lines show the corresponding analytical solutions $\mathbb {E}\big [K_\textrm{s}^{(M)}\big ]/R_{\textrm{s}}^{(3)}$ (see Eq. (20)). Dashed grey lines show the maximum possible relative overlaps $500/1500\approx 0.33$ and $750/1500=0.5$. **c** The Fano factor $\textrm{var}\big [K_\textrm{s}^{(M)}\big ]/{{\mathbb {E}}}\big [K_{\textrm{s}}^{(M)}\big ]$ associated with relative overlaps between individuals 1 and 2 (solid blue line), 2 and 3 (dash-dotted green line), 1 and 3 (dashed orange line), and 1–3 (solid red line) as a function of the number of sampled cells $S^{(m)}$ (Color figure online)

## Simulations

**Fig. 5:**
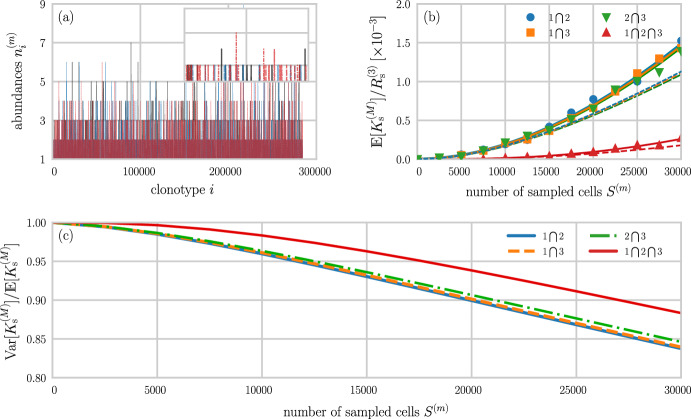
Sampling from empirical TRB CDR3 distributions and overlap measures in the number representation. **a** Distributions of TRB CDR3 cells in $M=3$ individuals. We used the SONIA package (Elhanati et al. 2014) to generate $10^{5}$ TRB CDR3 sequences for each individual. The sampled group richness $R_\textrm{s}^{(3)}$ was found to be 284, 598. Equal sample sizes $S^{(m)}$ were then drawn. **b** Relative overlaps between individuals 1 and 2 (blue disks), 2 and 3 (green inverted triangles), 1 and 3 (orange squares), and 1–3 (red triangles). The solid lines plot the corresponding analytical solutions $\mathbb {E}\big [K_\textrm{s}^{(M)}(\{\textbf{n}^{(m)}\})\big ]/R_{\textrm{s}}^{(3)}$ found in Eqs. (33). The dashed curves correspond to using using the estimator ${\hat{p}}_{i}^{(m)}= n_{i}^{(m)}/N^{(m)}$ in the expression $\mathbb {E}\big [K_{\textrm{s}}^{(M)}(\{\textbf{p}^{(m)}\})\big ]/R_\textrm{s}^{(3)}$ (Eq. (20) evaluated using $\rho _{i}^{(m)}(S)$ from Eq. (37)). **c** The Fano factor $\textrm{var}\big [K_\textrm{s}^{(M)}\big ]/{{\mathbb {E}}}\big [K_{\textrm{s}}^{(M)}\big ]$ associated with relative overlaps between individuals 1 and 2 (solid blue line), 2 and 3 (dash-dotted green line), 1 and 3 (dashed orange line), and 1–3 (solid red line) as a function of the number of sampled cells $S^{(m)}$ (Color figure online)

The sampling theory derived in the previous sections is useful for understanding the effect of different sampling distributions on measurable quantities such as the proportion of shared TCRs and BCRs among different individuals. Figures 4 and 5 show two examples of receptor distributions, along with the respective relative overlaps and Fano factors, for three individuals. To illustrate our methodology clearly and concisely, we utilize three shifted uniform distributions as models of synthetic sequence distributions in Fig. 4. In this example, the number of TCR or BCR sequences per individual is $N^{(m)}=10^{5}$, $(m=1,2,3)$, and the sampled group richness $R_{\textrm{s}}^{(3)}=1500$. Based on the abundance curves shown in Fig. 4a, we can readily obtain the overlaps between individuals 1–3 (solid black, dashed blue, and dash-dotted red lines), as well as between all pairs of individuals. The maximum possible overlap, normalized by $R_\textrm{s}^{(3)}$, between all three individuals and between individuals 1 and 3 is $500/1500\approx 0.33$. For the two remaining pairs, the corresponding maximum relative overlap, normalized by the richness associated with all three sampled individuals, is $750/1500=0.5$.

Using $S^{(m)}< N^{(m)}$ sampled cells from each individual, we observe in Fig. 4b that the increase of $\mathbb {E}[K_{\textrm{s}}^{(M)}]/R_{\textrm{s}}^{(3)}$ with $S^{(m)}$ is well-described by Eq. (20). In Fig. 4c, we plot the Fano factor $\textrm{var}[K_\textrm{s}^{(M)}]/{{\mathbb {E}}}[K_{\textrm{s}}^{(M)}]$ as a function of the number of sampled cells $S^{(m)}$ from each individual. For sample sizes of about 1000 (i.e., about 1% of the total sequence population), the Fano factor is between 0.4 (for overlaps between individuals 1 and 2 and between individuals 2 and 3) and 0.6 (for the overlap between individuals 1–3). As sample sizes reach about 5% of the total number of sequences ($10^{5}$), the variance $\textrm{var}[K_{\textrm{s}}^{(M)}]$ becomes negligible with respect to the expected overlap ${\mathbb {E}}[K_{\textrm{s}}^{(M)}]$.

As an example of an application to empirical TRB CDR3 data, we used the SONIA package (Elhanati et al. 2014) to generate amino acid sequence data for three individuals, each with $N^{(m)}=10^5$ cells. The combined richness across all individuals is $R_{\textrm{s}}^{(3)}=284,598$. We show the abundances of all sequences in Fig. 5a. The majority of sequences has an abundance of 1 while only very few sequences have abundances that exceed 5. Figure 5b shows the expected number of shared sequences as a function of the sampled number of cells $S^{(m)}$. To evaluate Eq. (33), we compute the binomial terms in ${\tilde{\sigma }}_{i}$ and ${\tilde{\sigma }}_{ij}$ by expanding them according to, e.g.,

45 $$\begin{aligned} \displaystyle \frac{{N^{(m)}-n_{i}^{(m)}\atopwithdelims ()S^{(m)}}}{{N^{(m)}\atopwithdelims ()S^{(m)}}} = \prod _{\ell =1}^{n_{i}^{(m)}}\left( 1-\frac{S^{(m)}}{N^{(m)}-n_{i}^{(m)}+\ell }\right) , \end{aligned}$$

where $S^{(m)}/N^{(m)}$ is the sample fraction drawn from the $m^{\textrm{th}}$ individual. For large $n_{i}$, other approximations, including variants of Stirling’s approximations can be employed for fast and accurate evaluation of binomial terms.

We compare these number-representation results with the $\textbf{p}$-representation results by using the estimates ${\hat{p}}_{i}^{(m)} = n^{(m)}_{i}/N^{(m)}$ in $\rho _{i}^{(m)}(S)$ and $\rho _{ij}^{(m)}(S)$ to compute the quantities in Eqs. (20) and (21). If the number of sampled cells $S^{(m)}$ is not too large, the analytic approximation of using ${\hat{p}}_{i}^{(m)}$ in $\rho ^{(m)}_{i}(S)$ to calculate $\mathbb {E}[K_\textrm{s}^{(M)}]/R_{\textrm{s}}^{(3)}$ is fairly accurate, as shown by the dashed curves in Fig. 5b. Since the abundances of the majority of sequences are very small, finite-size effects lead to deviations from the naive approximation (37) as the numbers of sampled cells $S^{(m)}$ grows large. Of course, we can also extract generation probabilities from SONIA and directly use Eq. (20) and $\rho _{i}^{(m)}(S)$ from Eq. (37) to find the $\textbf{p}$-representation *M*-overlap $\mathbb {E}[K_{\textrm{s}}^{(M)}(\{\textbf{p}^{(m)}\})]/R_{\textrm{s}}^{(3)}$.

To examine the variance associated with a given expected number of shared empirical TRB CDR3 sequences, we show the Fano factor $\textrm{var}[K_{\textrm{s}}^{(M)}]/{{\mathbb {E}}}[K_{\textrm{s}}^{(M)}]$ as a function of the sample size $S^{(m)}$ in Fig. 5c. For the shown sample sizes up to $S^{(m)}=3\times 10^{4}$, the Fano factor is larger than about 0.85, indicating a relatively large variance $\textrm{var}[K_\textrm{s}^{(M)}]$. In addition to reporting mean values of measures of sequence sharing (i.e., “overlap” or “publicness”) when analyzing empirical receptor sequence data (Elhanati et al. 2018; Ruiz Ortega et al. 2023), we thus recommend to compute $\textrm{var}[K_{\textrm{s}}^{(M)}]$ to determine corresponding confidence intervals.

Calculations were performed on an AMD^®^ Ryzen Threadripper 3970 using Numba to parallelize the calculation of Eqs. (33) and $\sum _{j\ne i}^{\Omega }\prod _{m=1}^{M}\rho _{ij}^{(m)}$ used in $\textrm{var}[K^{(M)}]$.

## Explicit Forms for Power-Law Probabilities

All of our results thus far assume a model or estimate of $p_{i}$ or $n_{i}$, as well as knowledge of at least $\Omega$. For our formulae to be useful, the theoretical maximum richness $\Omega$ also needs to be estimated or modeled. Numerous parametric and nonparametric approaches have been developed in the statistical ecology literature (Chao and Lee 1992; Wang and Lindsay 2005; Gotelli and Colwell 2011; Colwell et al. 2012; Gotelli and Chao 2013; Chiu et al. 2014; Chao and Lin 2012; Chao et al. 2020), as well as expectation maximization methods to self-consistently estimate richness and most likely clone population $\textbf{n}$ (Kaplinsky and Arnaout 2016).

**Fig. 6:**
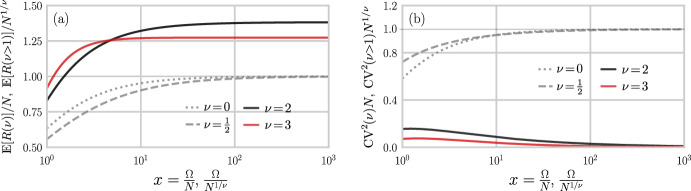
Expected richness and uncertainty under power law-distributed probabilities $p_{i}$ following Eq. (46). **a** Expected richness for different values of $\nu$ that lead to simple scaling and dependence only on $x=\Omega /N, \Omega /N^{1/\nu }$. For large *x*, the expected rescaled richnesses $\mathbb {E}[R(\nu )]/N$ and $\mathbb {E}[R(\nu >0)]/N^{1/\nu }$ converge. Since the normalization of the expected richness (by $N^{1/\nu }$) for $\nu > 1$ is different than for $\nu =0,\tfrac{1}{2}$, (normalized by *N*), $\mathbb {E}[R(\nu >0)]/N^{1/\nu }$ converges to greater values, but $\lim _{x\rightarrow \infty }\mathbb {E}[R(\nu >0)]$ remains $<1$. **b** From the variances $\mathbb {E}[R^{2}(\nu )]$, we construct the squared coefficient of variation and plot $\textrm{CV}^{2}N \equiv N\textrm{var}[R(\nu )]/(\mathbb {E}[R(\nu )])^{2}$ as a function of $x=\Omega /N$ (or $\textrm{CV}^{2}(\nu > 1)N^{1/\nu }$ for as a function of $x=\Omega /N^{1/\nu }$). For large *x*, $\textrm{CV}^{2}\approx 1/N$ for $\nu = 0,1/2$ but $\textrm{CV}^{2}N^{1/\nu }\sim 0$ for $\nu =2,3.$

To explore how our results depend on parameters such as $\Omega$, we derive approximate analytic expressions when the identical individual probabilities $p_{i}^{(m)}=p_{i}$ obey truncated power-law distributions:

46 $$\begin{aligned} p_{i} = \frac{i^{-\nu }}{H_{\nu }(\Omega )},\quad p_{j} \le p_{i}\,\,\, \text{ if } \nu \ge 0, \,\, j\le i, \,\,\, i,j = 1,2,\ldots , \Omega , \end{aligned}$$

where $H_{\nu }(\Omega )\equiv \sum _{j=1}^{\Omega } j^{-\nu }$. Such power laws are good approximations to measured ranked T cell clone abundances (Gaimann et al. 2020). If $\Omega$ is sufficiently large, we would like to show under what conditions the expectations of our diversity measures converge quickly to $\Omega$-independent values. By approximating $\sum _{i=1}^{\Omega }(1-p_{i})^{N} \approx \sum _{i=1}^{\Omega } e^{-Np_{i}} \approx \int _{1}^{\Omega } e^{-N/(H_{\nu }(\Omega )z^{\nu })}\text {d}z$ in Eq. (6) we find in the large $\Omega$ limit

47 $$\begin{aligned} \begin{aligned} \frac{\mathbb {E}[R(\nu =0)]}{N} \approx&\, x(1-e^{-1/x}),&x \equiv \Omega /N\qquad&\, \\ \frac{\mathbb {E}[R(\nu =\tfrac{1}{2})]}{N} \approx&\, x\left[ 1-2\text{ E }\left( 3,\tfrac{1}{2x}\right) \right] ,&x \equiv \Omega /N \qquad&\, \\ \frac{\mathbb {E}[R(\nu =1)]}{N} \approx&\, 1-\frac{\log N}{\log \Omega }+ \frac{\log (\log \Omega )}{\log \Omega },&\,&\, \\ \frac{\mathbb {E}[R(\nu >1)]}{N^{1/\nu }} \approx&\, x\left[ 1-\tfrac{1}{\nu }\text{ E }\left( 1+\tfrac{1}{\nu }, \tfrac{x^{-\nu }}{\zeta (\nu )}\right) \right] ,&x\equiv \Omega /N^{1/\nu } \,\,&\, \end{aligned} \end{aligned}$$

where the exponential integral is defined by $\text{ E }(x,y)\equiv \int _{1}^{\infty }\!t^{-x}e^{-yt}\text {d}t$ and $\zeta (\nu )$ is the Riemann zeta function. Consistent with known biology and previous estimates (Zarnitsyna et al. 2013; Lythe et al. 2016), we take the large-$\Omega$ limit where $x > 1$. From Eqs. (47), we see that the expected richnesses converge to fixed values for large enough $\Omega$ and all values of $\nu \not \approx 1$. The rescaled expected richnesses are plotted as functions of $x=\Omega /N$ or $x=\Omega /N^{1/\nu }$ in Fig. 6a.

Analogous cutoff-insensitive results can be found for the variance $\textrm{var}[R^{2}(\nu )]$ as well as other quantities. A good approximation for the variance is

48 $$\begin{aligned} \begin{aligned} \text{ var }[R(\nu )]&= \mathbb {E}[R^{2}(\nu )] - \left( \mathbb {E}[R(\nu )]\right) ^{2} \\ \,&\approx \sum _{i=1}^{\Omega } e^{-p_{i}N}\left( 1-e^{-p_{i}N}\right) \\ \,&\approx \tfrac{\Omega }{\nu }\left( \text{ E }\left( 1+\tfrac{1}{\nu }, \tfrac{N}{H_{\nu }(\Omega )\Omega ^{\nu }}\right) - \text{ E }\left( 1+\tfrac{1}{\nu }, \tfrac{2}{H_{\nu }(\Omega )\Omega ^{\nu }}\right) \right) , \end{aligned} \end{aligned}$$

where the power-law assumption in Eq. (46) is used in the last approximation. The normalized squared coefficients of variation $\text{ CV}^{2} \equiv \text{ var }[R(\nu )]/(\mathbb {E}[R(\nu )])^{2}$ for representative $\nu$ are found to be

49 $$\begin{aligned} \begin{aligned} \textrm{CV}^{2}_{\nu =0}N \approx&\, \frac{e^{-1/x}}{x(1-e^{-1/x})},&x \equiv \Omega /N\qquad \quad&\, \\ \textrm{CV}^{2}_{\nu =1/2}N \approx&\, \frac{2}{x} \frac{\textrm{E}(3,\tfrac{1}{2x}) -\textrm{E}(3,\tfrac{1}{x})}{\left( 1-2\textrm{E}(3,\tfrac{1}{2x})\right) ^{2}},&x \equiv \Omega /N\qquad \quad&\, \\ \textrm{CV}^{2}_{\nu =1}N \approx&\, \frac{\log \Omega \left[ \log (\Omega \log \Omega )-\log 4N\right] }{\left[ \log (\Omega \log \Omega )-\log N\right] ^{2}},&\,&\, \\ \textrm{CV}^{2}_{\nu > 1}N^{1/\nu } \approx&\, \frac{1}{\nu x}\frac{\textrm{E}\left( 1+\tfrac{1}{\nu }, \tfrac{x^{-\nu }}{\zeta (\nu )}\right) -\textrm{E}\left( 1+\tfrac{1}{\nu }, \tfrac{2x^{-\nu }}{\zeta (\nu )}\right) }{\left( 1-\tfrac{1}{\nu }\textrm{E}\left( 1+\tfrac{1}{\nu }, \tfrac{x^{-\nu }}{\zeta (\nu )}\right) \right) ^{2}},&x \equiv \Omega /N^{1/\nu } \qquad&\, \end{aligned} \end{aligned}$$

Plots of the CV of the richness under power-law system probabilities are shown in Fig. 6b. We see that the squared CVs converge in the large *x* limit to $N^{-1}$ for $\nu =0,1/2$ and vanish for $\nu > 1$.

The behavior of the sampled *M*-overlap, $\mathbb {E}[K_\textrm{s}^{(M)}(\{\textbf{p}^{(m)}\})]$, can also be quantified under the power-law probability distribution. By using Eq. (20) and $\rho _{i}(S)$ (Eq. (37), assuming equal probabilities $p_{i}^{(m)}=p_{i}$ and sample sizes $S^{(m)}=S$ across individuals), we find

50 $$\begin{aligned} \mathbb {E}\Big [K_{\textrm{s}}^{(M)}(\{\textbf{p}^{(m)}\})\Big ] = \sum _{i=1}^{\Omega }\prod _{m=1}^{M}\rho _{i}^{(m)}(S) \approx \sum _{i=1}^{\Omega }\left( 1-e^{-p_{i}S}\right) ^{M}. \end{aligned}$$

This expression can be further simplified in the large $\Omega$ limit for specific $\nu$,

51 $$\begin{aligned} \begin{aligned} \mathbb {E}[K_{\textrm{s}}^{(M)}(\nu =0)]&= \Omega \Big (1-(1-1/\Omega )^{S}\Big )^{M} \approx \Omega \left( 1-e^{-S/\Omega }\right) ^{M}, \\ \mathbb {E}[K_{\textrm{s}}^{(M)}(\nu \gtrsim 0.7)]&= \sum _{i=1}^{\Omega }\left( 1-(1-p_{i})^{S}\right) ^{M} \approx \sum _{i=1}^{\Omega }\exp \Big [-Me^{-\frac{Si^{-\nu }}{H_{\nu }(\Omega )}}\Big ] \\ \,&\sim \Big [1-e^{-\frac{S}{H_{\nu }(\Omega )}}\Big ]^{M} \left( \frac{S}{H_{\nu }(\Omega )\log M}\right) ^{1/\nu }\!\!\!\!\!, \quad S, \frac{S}{H_{\nu }(\Omega )}\gg 1. \end{aligned} \end{aligned}$$

The last approximation is most accurate for $\nu > 1$ where $H_{\nu }(\Omega \rightarrow \infty )$ converges and the prefactor in brackets is $\approx 1$. For sufficiently large *S*, it still provides a rough estimate of *M*-overlap for smaller values of $\nu$. Asymptotic expressions for even smaller values of $\nu$ can be found in the $S/H_{\nu }(\Omega ) \ll 1$ limit, but this limit yields very low expected *M*-overlap and is typically less informative.

**Fig. 7:**
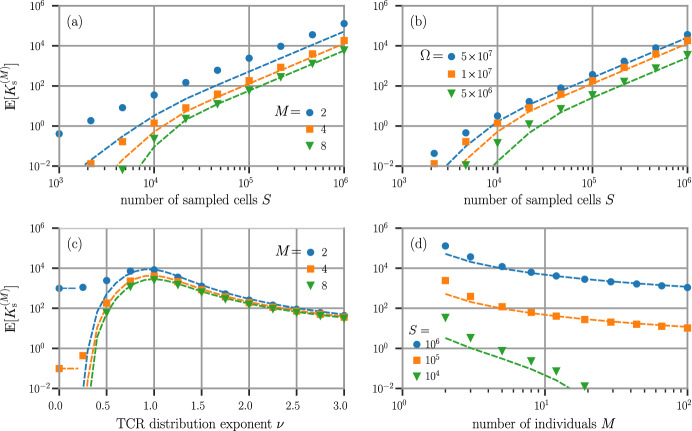
The expected *M*-overlap $\mathbb {E}[K_\textrm{s}^{(M)}(\{\textbf{p}^{(m)}\})]$. **a** Log-log plot of *M*-overlap as a function of individual sample size *S* using $p_{i} = i^{-1/2}/H_{1/2}(\Omega )$ ($\nu =1/2$) and $\Omega = 10^{7}$. $M=2,4,8$ are shown, with exponentially decreasing *M*-overlap as *M* is increased. **b** Fixing $M = 4$, a log-log plot of $\mathbb {E}[K_{\textrm{s}}^{(4)}(\nu =1/2)]$ against *S* for different values of $\Omega = 5\times 10^{6}, 10^{7}$ and $5\times 10^{7}$. **c** $\mathbb {E}[K_{\textrm{s}}^{(M)}(\nu )]$ plotted against $\nu$ for fixed $\Omega = 10^{7}$ and different *M*. (d) With $\Omega =10^{7}$, a log-log plot of $\mathbb {E}[K_\textrm{s}^{(M)}(\nu =1/2)]$ as a function of the number *M* of individuals sampled with $S=10^4, 10^{5}, 10^{6}$. In all panels, the dashed curves plot the analytic approximation for $\nu \gtrsim 0.7$ given in the second line of (51). In (c), the $\nu =0$ limit matches the expression given by the first line in (51). The approximations given in (51) are especially accurate for large *S* and larger *M* and $\nu$

Figure 7 plots the *M*-overlap $\mathbb {E}[K_\textrm{s}^{(M)}(\nu )]$ as a function of sample size, power-law $\nu$, and *M*. For comparison, the analytic approximation for $\nu \gtrsim 0.7$ (51) is also plotted by the dashed curves. Equation (51) and plots such as those in Fig. 7a, b could be useful for estimating the sample size *S* required in order to observe a specific overlap between the immune repertoires of *M* selected individuals. For instance, with $M=4$ individuals, a repertoire size of $\Omega =10^7$, and a sequence distribution exponent $\nu =0.5$, an expected *M*-overlap of approximately 1 can be achieved with a sample size of $S=10^4$.

Since the $\left( 1/H_{\nu }(\Omega )\right) ^{1/\nu }$ term in Eq. (51) increases with $\nu$, we expect that an effectively smaller repertoire size (recall $p_{i} \sim i^{-\nu }$ and larger $\nu$ leades to fewer larger-population clones), that the expected *M*-overlap increases with $\nu$. However, the $\left( S/\log M\right) ^{1/\nu }$ factor decreases with $\nu$ since larger *S* give rise to a larger number of ways clones sampled from different individuals can “avoid” each other. These features give rise to a maximum in $\mathbb {E}[K_{\textrm{s}}^{(M)}(\nu )]$, as shown in Fig. 7c.

Using Eqs. (33) and (38), we can also straightforwardly evaluate the variance of the *M*-overlap. For $\nu =0$ and uniform $p_{i} = 1/\Omega$, $\text{ var }[K_\textrm{s}^{(M)}(\nu =0)]\approx \Omega (1-e^{-S/\Omega })^{M}\left( 1-(1-e^{-S/\Omega })^{M}\right)$. We find the variances of the *M*-overlap, as with our other metrics, are well approximated by that of a binomial process in the $S \rightarrow \infty$ limit and when values of $\nu$ are modest: $\text{ var }[K_{\textrm{s}}^{(M)}]\approx \Omega \tfrac{\mathbb {E}[K_\textrm{s}^{(M)}]}{\Omega }\Big (1-\tfrac{\mathbb {E}[K_\textrm{s}^{(M)}]}{\Omega }\Big )$. Modest deviations from this approximation arise for finite *S* and large values of $\nu$.

## Sampling Resolution and Information Loss

We end with a brief discussion of information loss upon coarse-graining which arises when analyzing lower-dimensional experimental/biochemical classifications of clones that are commonly used. Such lower-dimensional representations can be obtained through spectratyping (Gorski et al. 1994; Fozza et al. 2017). For TCRs, spectratyping groups sequences together and produces compressed receptor representations describing CDR3 length, frequency, and associated beta variable (TRBV) genes (Gkazi et al. 2018). In addition to coarse-grained representations of sequencing data, some studies (Elhanati et al. 2018; Ruiz Ortega et al. 2023) use continuous approximations to describe the distribution of receptor sequences. Estimators of entropy and their errors have been developed for subsampling from discrete distributions (Schürmann 2004; Grassberger 2022). Therefore, in this section, we focus on quantifying differences in information content that are associated with (i) using continuous approximations of discrete sequencing data, and (ii) coarse-graining already-discretized (i.e., spectratyping) distributions.

Given a discrete random variable *X* describing $\Omega$ “traits” and taking on possible values $\{x_1,x_2,\dots ,x_{\Omega }\}$, let $p_i=\mathbb {P}(X=x_i)$. The entropy of this probability distribution is given by $H_p=-\sum _{i=1}^{\Omega } p_i \log p_i$. Similarly, one might define the differential entropy for a continuous random variable taking on values in the interval [*a*, *b*] as $S_{p} = -\int _a^b p(x) \log p(x) \ \textrm{d} x$. It is well-known that the differential entropy is not a suitable generalization of the entropy concept to continuous variables (Jaynes 1963) since it is not invariant under change of variables and can be negative. These issues can be circumvented by introducing the limiting density of discrete points. Here, we present a more direct approach that will be sufficient for our application. For a probability density function $p:\ [a,b] \rightarrow {\mathbb {R}}_0^+$ we introduce a discretizing morphism ${\mathcal {D}}_{\Delta }$ so that

52 $$\begin{aligned} q_i = \int \nolimits _{a+(i-1)\Delta }^{a+i\Delta } p(x) \textrm{d} x, \quad i = 1,2,\dots , B. \end{aligned}$$

describes a random variable taking on values in each of the $(b-a)/\Delta = B$ bins.

To quantify the amount of information lost in this discretization step, consider the entropy $H_q = -\sum _{i=1}^{B}q_{i}\log q_{i} \sim \log \Delta$ in the $\Delta \rightarrow 0$ limit.

**Fig. 8:**
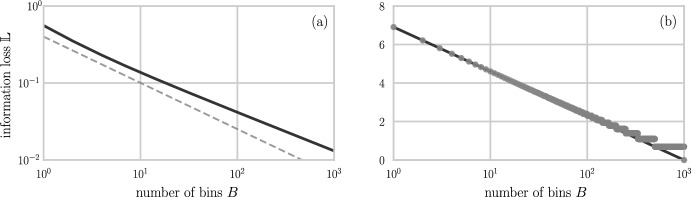
The information loss ${\mathbb {L}}$ as a function of the number of discretization bins *B*. The loss is least as the number of integration bins $B\rightarrow \infty$. **a** The solid black line shows the information loss associated with discretizing a truncated power law (see Eq. (54)), and the dashed grey line is a guide-to-the-eye (power law) with slope $-0.6$. **b** Grey dots show the information loss associated with coarse graining a discrete and uniform random variable with initially $\Omega =1000$ traits. The solid curve shows the corresponding analytical result for the difference in information ${\mathbb {L}} = -\log (B/\Omega )$ between discretizing a continuous uniform distribution of $\Omega =1000$ traits using *B* bins

If we want to evaluate any information loss as a difference between the (finite) differential entropy $S_{p}$ and the (diverging) entropy $H_{q}$ we need to account for this logarithmic contribution by defining the corresponding information loss as

53 $$\begin{aligned} {\mathbb {L}}(\Delta ) = \vert (S_{p}- \log \Delta ) - H_q\vert . \end{aligned}$$

By absorbing the logarithmic contribution into the differential entropy, we find the correct continuous entropy according to Jaynes (1963) using the limiting density of discrete points.

As an example, we compute the information loss associated with discretizing the truncated power law

54 $$\begin{aligned} p(x) = \left\{ \begin{array}{ll} \frac{1}{\gamma (\tfrac{1}{2},1)} \frac{e^{-x}}{\sqrt{x}}, &{} \text {if}\quad 0\le x \le 1 \\ 0, &{} \text {else} \end{array}\right. , \end{aligned}$$

where $\gamma (s,x)=\int _{0}^{x} t^{s-1} e^{-t}\,\textrm{d}t$ is the lower incomplete gamma function. The distribution Eq. (54) gives rise to few high-abundance clones and many low-abundance clones, as typical for TCR receptor sequences (Xu et al. 2020). Analytic expressions for the discretized probabilities $q_i$ are lengthy, so we numerically compute $q_i$ to evaluate the information loss ${\mathbb {L}}(\Delta )$. Equation (53) is plotted as a function of the number of bins $B=1/\Delta$ in Fig. 8a. The information loss decreases with the number of bins, as this results in the discrete distribution gathering more information about its continuous counterpart.

While the connection between continuous probability distributions and their discretized counterparts has important consequences for sampling, information loss also occurs in spectratyping when an already discrete random variable is coarse grained. In this scenario, the information loss can be quantified uniquely (up to a global multiplicative constant) by the entropy difference of the distributions (Baez et al. 2011). The difference between the full $H_p=-\sum _{i=1}^{\Omega } p_i \log p_i$ and the coarse-grained $H_q = -\sum _{i=1}^{B}q_{i}\log q_{i}$ can be explicitly evaluated for uniformly distributed probabilities.

For any number $B<\Omega$ we can define a coarse graining procedure that yields only *B* traits by defining the bin size $\Delta ={\text {ceil}}(\Omega /B)$ and grouping together $\Delta$ traits into each bin. The last bin might be smaller than the other bins or even empty. The information loss of this procedure is shown in Fig. 8b for an initially uniform distribution of $\Omega = 1000$ traits. Across certain ranges of *B*, plateaus can build since our coarse graining might add zero probabilities. However, we can instead start from a continuous distribution and compare the discretization with $\Omega =1000$ bins to any other binning with $B\le \Omega$.

Comparing a coarse-grained uniform distribution with *B* bins to the discretized distribution with $\Omega$ bins yields the information loss with respect to the initial discrete distribution ${\mathbb {L}} = -\log (B/\Omega ) \ge 0$. We plot this analytical prediction against the information loss ${\mathbb {L}}$ associated with coarse graining an already discrete distribution in Fig. 8b, showing them to be well-aligned.

## Discussion and Conclusions

Quantifying properties of cell-type or sequence distributions is an important aspect of analyzing the immune repertoire in humans and animals. Different methods have been developed to estimate TCR and BCR diversity indices such as the total number of distinct sequences in an organism (i.e., species richness) (Rempala and Seweryn 2013; Kaplinsky and Arnaout 2016; Xu et al. 2020). Another quantity of interest is the number of clones that are considered “public” or “private,” indicating how often certain TCR or BCR sequences occur across different individuals.

Public TCR$\beta$ and BCR sequences have been reported in a number of clinical studies (Putintseva et al. 2013; Robins et al. 2010; Shugay et al. 2013; Soto et al. 2020; Briney et al. 2019; Soto et al. 2019). However, the terms “public” and “private” clonotypes are often based on different and ambiguous definitions. According to Shugay et al. (2013), a “public sequence” is a sequence that is “*often* shared between individuals” (Shugay et al. 2013), while Greiff et al. (2017) refers to a sequence as public if it is “shared across individuals”. In addition to ambiguities in the definition of what constitutes a private/public sequence, overlaps between the immune repertoires of different individuals are often reported without specifying confidence intervals, even though variations may be large given small sample sizes and heavy tailed sequence distributions.

In this work, we provided mathematical definitions for “public” and “private” clones in terms of the probabilities of observing a number of clones across *M* selected individuals, complementing related work that introduced the notion of “sharing number *M*” (i.e., the expected number of sequences which will be found in *exactly* *M* individuals) to quantify the expected overlap between cell-sequence samples (Elhanati et al. 2018; Ruiz Ortega et al. 2023). Besides defining individual repertoire probability distributions, our results include analytic expressions for individual and multi-individual expected richness and expected overlap as given by Eqs. (5), (10), (17), (19), and (20). Additionally, using Eqs. (28) to (33), we derived expressions for the expected richness and expected overlap in subsamples. The variability of quantities (second moments) such as the *M*-overlap and subsampled overlap were also derived. Studies analyzing the similarities and differences associated with immune repertoires of different individuals (see, e.g., Elhanati et al. 2018; Soto et al. 2020; Ruiz Ortega et al. 2023) may utilize our results on second moments of overlap measures to quantify the statistical significance of their findings. Our results are summarized in Table 1 where we provide expectations and second moments of all quantities as a function the cell population configurations $\textbf{n}^{(m)}$ or as a function of the underlying clone generation probabilities $\textbf{p}^{(m)}$, as is generated by models such as SONIA (Elhanati et al. 2014).

Further inference of richness and overlap given sample configurations can be developed using our results. For example, the parametric inference of expected richness in an individual given a sampled configuration $\textbf{s}$ can be found using the multinomial model and Bayes’ rule, as presented in Eq. (44).

While our results depend on knowledge of $\Omega$ and *N*, we show using power-law probability distributions and explicit expressions in Eqs. (47) and (49) that the richness is insensitive to $\Omega$ in the large *N* and $\Omega /N$ limits. Therefore, even though $\Omega$ may be impossible to accurately estimate, power-law probability distributions generally render our results robust to uncertainty in $\Omega$. Analytic or semi-analytic expressions for the overlap quantities can also be derived. We leave this exercise to the reader.

Finally, in the context of coarse-graining, or spectratyping (Ciupe et al. 2013), we have discussed methods that are useful to quantify the information loss associated with different levels of coarse graining TCR and BCR sequences. The results presented here are based on an assumption of simple multinomial distributions as the underlying population model. A number of mechanistically more realistic probability distributions have been derived for neutral, noninteracting clone populations in steady state (Dessalles et al. 2018). These include log series and negative binomial distributions each requiring tailored calculations for the corresponding richness and overlap.

## Data Availability

All source codes are publicly available at https://gitlab.com/ComputationalScience/immune_repertoires.
